# Effect of higher dose primaquine for the radical cure of *Plasmodium vivax* malaria in Indonesia: a systematic review and individual patient data meta-analysis

**DOI:** 10.1016/j.lanwpc.2026.101908

**Published:** 2026-06-18

**Authors:** Ihsan Fadilah, James A. Watson, Ayodhia P. Pasaribu, Inge Sutanto, Erni J. Nelwan, Kartini Lidia, Megha Rajasekhar, Iqbal RF. Elyazar, Walter RJ. Taylor, Kamala Thriemer, Nicholas PJ. Day, Jeanne Rini Poespoprodjo, Julie A. Simpson, Ric N. Price, J. Kevin Baird, Robert J. Commons

**Affiliations:** a Oxford University Clinical Research Unit Indonesia, Faculty of Medicine Universitas Indonesia, Jakarta, Indonesia; b Nuffield Department of Medicine, Centre for Tropical Medicine and Global Health, University of Oxford, Oxford, United Kingdom; c Infectious Diseases Data Observatory, Nuffield Department of Medicine, Centre for Tropical Medicine and Global Health, University of Oxford, Oxford, United Kingdom; d Department of Pediatrics, Medical Faculty, Universitas Sumatera Utara, Medan, Indonesia; e Yayasan Penguatan Kesehatan Masyarakat Tridarma (YPKMT)/Tridarma Healthcare Empowerment Foundation (THEMP), Medan, Indonesia; f Department of Parasitology, Faculty of Medicine, Universitas Indonesia, Jakarta, Indonesia; g Faculty of Medicine, Universitas Indonesia, Jakarta, Indonesia; h Division of Tropical Medicine and Infectious Disease, Department of Internal Medicine, Cipto Mangunkusumo Hospital, Jakarta, Indonesia; i Department of Pharmacology and Therapy, Faculty of Medicine and Veterinary Medicine, Universitas Nusa Cendana, Kupang, Indonesia; j Centre for Epidemiology and Biostatistics, Melbourne School of Population and Global Health, The University of Melbourne, Melbourne, VIC, Australia; k Mahidol-Oxford Tropical Medicine Research Unit, Faculty of Tropical Medicine, Mahidol University, Bangkok, Thailand; l Global and Tropical Health Division, Menzies School of Health Research and Charles Darwin University, Darwin, Australia; m Centre for Child Health, Faculty of Medicine, Public Health and Nursing, Universitas Gadjah Mada, Yogyakarta, Indonesia; n Yayasan Pengembangan Kesehatan dan Masyarakat Papua, Timika, Papua, Indonesia; o General and Subspecialty Medicine, Grampians Health Ballarat, Ballarat, Australia

**Keywords:** Primaquine, *Plasmodium vivax*, Malaria, Relapse, Efficacy, Safety, Tolerability, Indonesia

## Abstract

**Background:**

*Plasmodium vivax* malaria has diverse transmission and relapse patterns in Indonesia. The optimal dose of primaquine to prevent relapses across the country is unknown. We evaluated the anti-relapse efficacy, gastrointestinal tolerability, and haematological safety (G6PD activity ≥30%) of different primaquine regimens in varied endemic settings in Indonesia.

**Methods:**

We systematically searched for studies published between 1 January 2000 and 23 July 2024 prospectively enrolling patients with acute uncomplicated *P. vivax* malaria where some patients were treated with primaquine. Individual patient data (IPD) from eligible studies were pooled and harmonised. We fitted one-stage IPD multivariable regression models to estimate the causal relationship between the body weight-adjusted primaquine dose with three separate primary outcomes: (i) the time to first *P. vivax* recurrence (days 7–180), (ii) any gastrointestinal discomfort (days 5–7), and (iii) ≥25% reduction relative to baseline haemoglobin and a reduction to <7 g/dL (days 1–14).

**Findings:**

Of ten eligible studies, seven were available for inclusion. Compared with a total dose of 3.5 mg/kg primaquine, patients treated with a total dose of 7 mg/kg had a lower rate of recurrence over 6 months (adjusted hazard ratio 0.53; 95% confidence interval [CI] 0.45–0.63; n = 1797); the relative efficacy was consistent across regions, but the absolute benefit varied. Gastrointestinal discomfort was more frequent with higher doses (adjusted risk ratio 1.32 per 0.25 mg/kg daily dose; 95% CI 1.15–1.51; n = 952). In 822 patients available to be assessed for haematological safety (788 [96%] with G6PD activity ≥70% and 34 [4%] with activity 30% to <70%), only one patient developed clinically relevant haemolysis.

**Interpretation:**

Across all transmission settings in Indonesia, a total dose of 7 mg/kg halved the rate of recurrent *P. vivax* malaria over a 6-month period compared with the low dose of 3.5 mg/kg. However, increased daily doses slightly increased risks of gastrointestinal discomfort and haemolysis.

**Funding:**

NDM Tropical Network Fund, Bill and Melinda Gates Foundation.

**Research in context**

**Evidence before this study**

Indonesia is a geographically vast country with heterogeneous patterns of periodicity and varying magnitudes of risk for *Plasmodium vivax* relapse. However, the optimal regimen for the radical cure of *P. vivax* is unknown. The national antimalarial guidelines continue to recommend a low-dose regimen of 3.5 mg/kg without routine glucose-6-phosphate dehydrogenase (G6PD) testing. We systematically searched for trials conducted in Indonesia that randomly assigned patients with *P. vivax* malaria to primaquine dosing regimens. Articles published in any language between 1 January 2000 and 23 July 2024, using the terms “vivax” and “primaquine” in MEDLINE, Embase, Web of Science, Scopus, and the Cochrane Library were identified. No published randomised controlled trial directly comparing high-dose (≥5 mg/kg total) versus low-dose (2 to <5 mg/kg total) primaquine in Indonesia was identified. We identified two trials that randomised patients with *P. vivax* malaria to receive either high-dose (target total dose of 7 mg/kg over seven or 14 days) or low-dose (3.5 mg/kg over 14 days) primaquine, compared with placebo, in combination with dihydroartemisinin-piperaquine. One multi-country trial included two study sites in Sumatra: Hanura and Tanjung Leidong. Compared with placebo, high-dose primaquine reduced relapse by 60–89%. In another trial, patients acquired infections in Papua and were followed in malaria-free East Java. Low-dose primaquine reduced relapse by 74% compared with placebo.

**Added value of this study**

This country-specific systematic review and individual patient data meta-analysis included 1797 patients from seven studies evaluating anti-relapse efficacy, gastrointestinal tolerability, and haematological safety. The analysis represents the most comprehensive analysis to date of different primaquine dosing regimens for *P. vivax* malaria in Indonesia. The results suggest that doubling the total dose from 3.5 mg/kg to 7 mg/kg would halve the rate of *P. vivax* recurrence within 180 days, however, the absolute benefit of this reduction depends on the underlying risk of recurrence in different regions of Indonesia. An increase in the daily dose of primaquine resulted in a moderate increase in gastrointestinal discomfort; and only a rare occurrence of clinically relevant haemolysis, given that G6PD activity among patients was mostly ≥70%.

**Implications of all the available evidence**

For Indonesian patients with *P. vivax* malaria, a high total primaquine dose of 7 mg/kg is more efficacious than the currently implemented 3.5 mg/kg regimen. Higher daily doses of primaquine are associated with increased gastrointestinal discomfort, however, these may be mitigated by co-administration with food. Clinically relevant haemolysis is rare in patients with G6PD activity >30%.

## Introduction

*Plasmodium vivax* is more geographically widespread than other causes of human malaria. In regions where *P. vivax* and *Plasmodium falciparum* are co-endemic, *P. vivax* is increasingly the predominant species because conventional malaria control efforts more readily impact *P. falciparum*.^1^ As of 2024, nearly 80% of districts in Indonesia had achieved malaria-free status.^2^ However, *P. vivax* malaria remains prevalent with over 100,000 reported cases in recent years; most of them from the eastern Papua region.^3^^,^^4^

Dormant liver stages (hypnozoites) of *P. vivax* cause repeated blood-stage infections (relapses) and attacks of acute malaria that constitute a major cause of morbidity and mortality, particularly among young children and pregnant women.^5^ Relapsing *P. vivax* avoids conventional control efforts aimed at treating acute malaria and minimising mosquito vector contact. Radical cure of acute malaria, by combining blood schizontocidal drugs with an 8-aminoquinoline (primaquine or tafenoquine) that kills dormant hypnozoites, remains the only effective strategy to avert *P. vivax* relapses.

A meta-analysis of malaria studies across *P. vivax*-endemic regions globally published in 2024 suggested that a high total dose of 7 mg/kg can halve the rate of *P. vivax* recurrences compared with a low total dose of 3.5 mg/kg, with only a slight increase in gastrointestinal discomfort.^6^ Patients treated with a daily primaquine dose of up to 0.5 mg/kg with glucose-6-phosphate dehydrogenase (G6PD) activity >30% had comparable risks of haemolysis with patients not exposed to primaquine.^7^

Updated 2024 WHO guidelines reflected these findings, with a high total dose of primaquine recommended over either 14 days or 7 days.^8^ Indonesian national guidelines currently recommend a low-dose regimen of 3.5 mg/kg total over 14 days without routine G6PD testing, with a higher dose (7 mg/kg over 14 days) recommended for cases of suspected relapse or treatment failure.^9^ This conservative dosing strategy reflects ongoing operational challenges in implementing universal G6PD testing at scale within programme settings.^10^ However, Indonesia is a geographically large country with varied *P. vivax* epidemiological characteristics. For example, in Papua, Indonesia, there is a high rate of *P. vivax* transmission and high risk of relapse, even when high total dose primaquine is given,^11^, ^12^, ^13^ while other regions of Indonesia have reported good efficacy rates with low dose primaquine.^14^^,^^15^ Thus, the potential heterogeneity in effects, as well as the overall risks and benefits of different primaquine regimens for Indonesia, remains unclear. Currently, there is limited evidence from individual-level, head-to-head comparisons of primaquine regimens across regions in the country.

This study aimed to assess the anti-relapse efficacy, gastrointestinal tolerability, and haematological safety of the different primaquine doses in patients with *P. vivax* malaria in Indonesia.

## Methods

### Search strategy and selection criteria

Building on an existing living systematic review^16^ of studies of patients with acute uncomplicated *P. vivax* malaria, we conducted an updated systematic search of MEDLINE, Embase, Web of Science, Scopus, and the Cochrane Library for studies conducted in Indonesia and published in any language between 1 January 2000 and 23 July 2024. Search terms are provided in Supplementary Material (List S1). Two reviewers (IF and RJC) conducted the review and resolved discrepancies through discussion, with JAW involved in cases of disagreement. The protocol was pre-registered with PROSPERO (CRD42024580630). We included randomised or non-randomised therapeutic trials with ≥28 days of active follow up for gastrointestinal tolerability and haematological safety, or ≥42 days for anti-relapse efficacy. Eligible studies included at least one group receiving multi-day primaquine for relapse prevention, initiated within seven days of blood schizontocidal treatment (chloroquine, quinine, or common artemisinin-based combination therapies).

### Data pooling

Investigators of eligible studies were invited to contribute individual patient data (IPD), including unpublished data when possible. Shared data were uploaded to the Worldwide Antimalarial Resistance Network repository and curated in accordance with the Infectious Diseases Data Observatory (IDDO) SDTM Implementation Guide.^17^ Patients were excluded if key variables were missing (age, sex, weight, baseline parasitaemia, treatment regimen), they had severe malaria, pregnancy, mixed infections, or received additional antimalarials after schizonticidal treatment. Additional inclusion criteria are described in Supplementary Material (List S2). This study analysed pseudo-anonymised data from IDDO. Original data controllers ensured datasets were collected with applicable ethical approvals and informed consent. IDDO performed additional de-identification during curation to ensure pseudonymisation before release, complying with UK GDPR, EU GDPR, and the Data Protection Act 2018. This IPD meta-analysis met the Oxford Tropical Research Ethics Committee criteria for waiving ethical review and patient informed consent, as it is a secondary analysis of existing pseudonymised data without new patient recruitment or interventions. This study is reported according to PRISMA-IPD guidelines^18^ (Table S1).

### Endpoints

Day zero was defined as initiation of antimalarial treatment after enrolment. The primary efficacy endpoint was the time to the first *P. vivax* recurrence (ascertained by microscopy), irrespective of symptoms, between days 7–180. Secondary endpoints included the time to the first symptomatic recurrence and the number of recurrences within the same timeframe. The primary gastrointestinal tolerability endpoint was a composite endpoint including any of the following signs of gastrointestinal discomfort, ascertained by symptom questionnaire, occurring between days 5–7 (after the resolution of acute malaria symptoms and completion of schizonticidal treatment): vomiting, anorexia, diarrhoea. Secondary tolerability endpoints included similar symptoms on day 0 and days 1–2, and acute vomiting within 1 h of primaquine administration. The primary haematological safety endpoint was a ≥25% haemoglobin reduction from baseline to <7 g/dL between days 1–14 (HemoCue™ system). Secondary endpoints included (i) maximum haemoglobin drop between days 1–14, 2–3, and 5–7, (ii) incident anaemia (Hb < 11 g/dL) during days 2–3 in patients with baseline Hb ≥ 11 g/dL and normal G6PD activity (≥70% activity^19^; study subgroup of interest), and (iii) severe haemolytic adverse events (Hb < 5 g/dL, >5 g/dL drop, blood transfusion, renal failure requiring dialysis, or death) within 14 days.

### Exposures

The efficacy exposure was the weight-adjusted (i.e., mg of primaquine base per kg of body weight [mg/kg]) total primaquine dose. The gastrointestinal tolerability and haematological safety exposure was the weight-adjusted daily dose.^6^^,^^7^ Doses were calculated from daily tablets or the mg administered and if these were unavailable they were inferred from protocols. Primaquine dose was specified in our models as a continuous variable. The effects of different categories of primaquine dose (List S2) were explored.

### Data analysis

The main analyses estimated the effect of primaquine dose on recurrence, gastrointestinal tolerability, and haemolysis under a one-stage IPD meta-analysis framework.

#### Anti-relapse efficacy

Cumulative incidence of first recurrence was modelled with a multivariable Cox proportional hazards model including total primaquine dose, age, sex, natural log-transformed baseline parasite density, and study site. Age and sex were each further handled to account for non-proportional hazards: age through an interaction term with follow-up time, and sex through stratification. Study site (n = 6; Timika, Tanjung Leidong, Lumajang, Kupang, Sragen, Hanura) was specified as a stratification variable to allow each site to have its own baseline hazard function. A causal directed acyclic graph (DAG) guided model development (Figure S1), and a restricted cubic spline for total primaquine dose allowed for nonlinearity (See List S2 for further methodological details). We evaluated potential differential effects of total primaquine dose by age group (<5 years or ≥5 years) and origin of infection (within or outside Papua; reflecting a higher potential burden of hypnozoites and/or more primaquine-tolerant strains within Papua). Additional analyses, including sensitivity analyses and assessment of residual confounding using negative controls are described in Supplementary Material (List S2).

#### Gastrointestinal tolerability

We summarised the outcome prevalence descriptively across study sites to highlight heterogeneity in the measurement of this endpoint. In the randomised subset of these data, the risk of gastrointestinal discomfort was modelled using multivariable Poisson regression^20^ with daily primaquine dose, age, sex, and log baseline parasite density (based on a DAG; Figure S3), with dose categories and splines used to assess nonlinear effects. Study site was not included in the model, as the study population for this outcome originated from a single trial conducted under the same protocol. Similar model specification was used to model the risk of acute vomiting. As the outcome prevalence was high, Poisson regression was used to model the binary outcomes to estimate the risk ratio. Effect estimates from modified Poisson models^20^ were obtained as part of the sensitivity analysis.

#### Haematological safety

The presence of clinically significant haemolysis (≥25% haemoglobin reduction from baseline to <7 g/dL) between days 1–14 was described. Multivariable linear regression was used to model the maximum haemoglobin drop from baseline over 14 days, including as covariates: daily primaquine dose, baseline haemoglobin, log_2_ G6PD activity, age, sex, baseline parasite density, study site, and a dose-sex-log_2_ G6PD interaction. A DAG guided model specification (Figure S4), and splines were used to assess nonlinear effects. Additional analyses, including a subgroup analysis of incident anaemia and analysis of methaemoglobinaemia, are described in Supplementary Material (List S2).

Risk of bias was assessed using the ROB2 (randomised trials)^21^ and ROBINS-I tools (non-randomised studies),^22^ adapted for the current study objectives. Statistical analysis was performed using R (v4.3.0) according to a pre-specified plan.^23^ We conducted posthoc analyses presented in Discussion, including an aggregate meta-analysis of trials directly comparing high and low total doses outside Indonesia, to strengthen our findings.

### Role of the funding source

The study funder had no role in study design, data collection, analysis, interpretation, and manuscript writing.

## Results

**Fig. 1:**
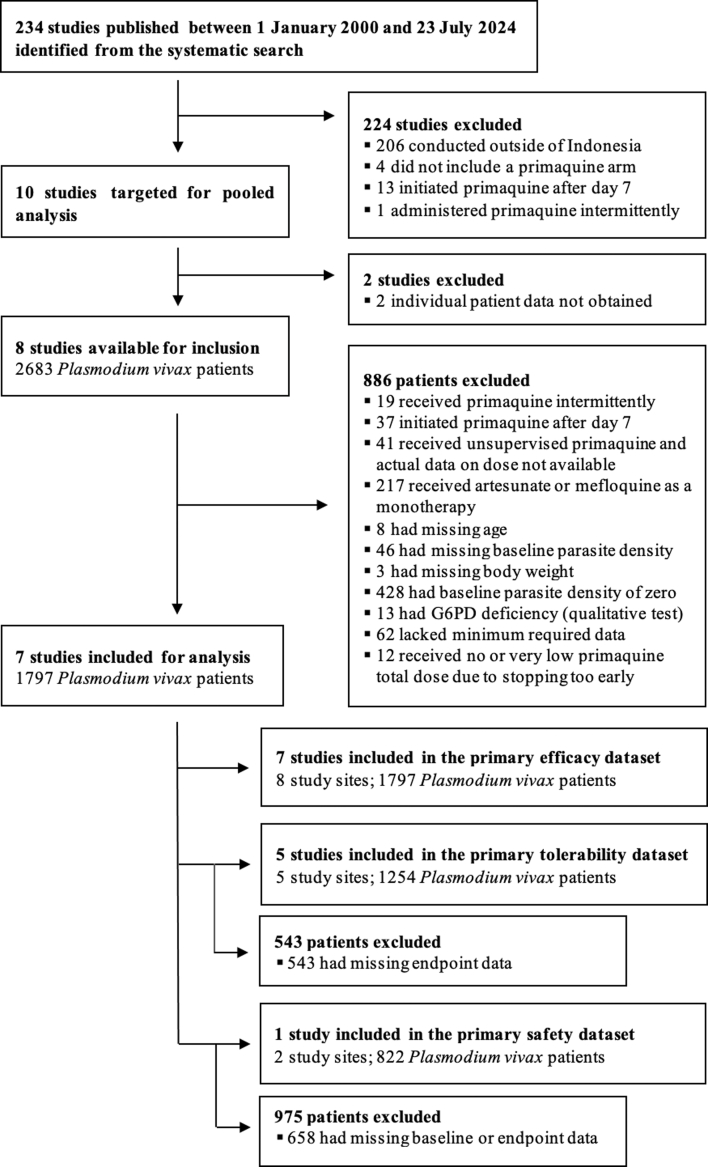
**Study and patient selection.** Databases systematically searched included MEDLINE, Embase, Web of Science, Scopus and the Cochrane Library. Additional analyses (e.g., sensitivity or restricted analyses) were conducted on smaller subsets of the relevant primary dataset. Forty-one patients were excluded as protocol-based dose calculation was considered unreliable in the context of unsupervised primaquine. Missing endpoint data in the tolerability and safety datasets primarily reflects studies that did not systematically measure the outcome of interest.

Between 1 January 2000 and 23 July 2024, 234 *P. vivax* efficacy studies were published, of which ten included Indonesian study sites and were eligible for the pooled analysis. IPD from eight studies were available for analysis. After patient-level exclusion criteria were applied, 1797 patients enrolled into seven studies were included in the analysis (Table 1 and Fig. 1). The seven studies included one trial that individually randomised patients to different primaquine dose regimens,^15^ while the remaining six administered the same total primaquine dose within each study (a target total of either 3.5 or 7 mg/kg).^11^, ^12^, ^13^, ^14^^,^^27^^,^^28^ There was a low to moderate risk of within study bias (Table S2).

**Table 1:** Patient characteristics by study.

Characteristic	Study							Overall
	Hasugian et al. 2007^27^	Pasaribu et al. 2013^14^	Sutanto et al. 2013^13^	Lidia et al. 2015^28^	Nelwan et al. 2015^11^	Taylor et al. 2019^15^	Poespoprodjo et al. 2022^12^	
Number of patients	139	331	39	51	118	963	156	1797
Median age (Q1, Q3; years)	13 (4, 26)	14 (9, 27)	27 (25, 29)	33 (25, 41)	28 (25, 31)	16 (10, 29)	16 (7, 29)	17 (10, 29)
Age group								
Less than 5 years	44 (32%)	26 (8%)	0 (0%)	0 (0%)	0 (0%)	69 (7%)	33 (21%)	172 (9%)
5–14 years	29 (21%)	148 (45%)	0 (0%)	0 (0%)	0 (0%)	386 (40%)	42 (27%)	605 (34%)
At least 15 years	66 (47%)	157 (47%)	39 (100%)	51 (100%)	118 (100%)	508 (53%)	81 (52%)	1020 (57%)
Male sex	73 (53%)	186 (56%)	39 (100%)	24 (47%)	118 (100%)	534 (55%)	82 (53%)	1056 (59%)
Body weight (kg)	43 (14, 53)	38 (22, 52)	64 (59, 72)	42 (36, 45)	69 (63, 74)	44 (24, 55)	45 (19, 56)	45 (24, 57)
Baseline parasite density (per μL)	1575 (375, 5244)	760 (320, 3100)	2848 (784, 4432)	2640 (1500, 6560)	880 (148, 2496)	3911 (1170, 8659)	4920 (1680, 11,550)	2793 (630, 7130)
Presence or recent history of fever	139 (100%)	321 (97%)	6 (16%)	51 (100%)	NA	850 (88%)	156 (100%)	1523 (91%)
Number of missing data	0	0	2	0	118	0	0	120
Schizonticidal drug								
Dihydroartemisinin-Piperaquine	68 (49%)	164 (50%)	0 (0%)	25 (49%)	58 (49%)	961 (100%)	156 (100%)	1432 (80%)
Artesunate-Amodiaquine	71 (51%)	167 (50%)	0 (0%)	0 (0%)	0 (0%)	0 (0%)	0 (0%)	238 (13%)
Artesunate-Pyronaridine	0 (0%)	0 (0%)	0 (0%)	0 (0%)	60 (51%)	0 (0%)	0 (0%)	60 (3.3%)
Chloroquine	0 (0%)	0 (0%)	0 (0%)	26 (51%)	0 (0%)	2 (0.2%)	0 (0%)	28 (1.6%)
Quinine	0 (0%)	0 (0%)	39 (100%)	0 (0%)	0 (0%)	0 (0%)	0 (0%)	39 (2.2%)
Primaquine total dose (mg base/kg)	3.8 (3.4, 4.3)	4.2 (3.6, 4.8)	7.1 (6.7, 8.2)	4.5 (4.2, 4.9)	7.1 (6.5, 8.1)	7.0 (6.0, 7.9)	7.3 (5.9, 8.2)	6.4 (4.1, 7.5)
Primaquine total dose group								
No primaquine (0 mg base/kg)	0 (0%)	0 (0%)	0 (0%)	0 (0%)	0 (0%)	196 (20%)	0 (0%)	196 (11%)
Low (≥2 and < 5 mg base/kg)	118 (85%)	265 (80%)	0 (0%)	39 (76%)	1 (0.85%)	21 (2.2%)	20 (13%)	464 (26%)
High (≥5 mg base/kg)	21 (15%)	66 (20%)	39 (100%)	12 (24%)	117 (99%)	746 (77%)	136 (87%)	1137 (63%)
Primaquine daily dose (mg base/kg)	0.27 (0.24, 0.31)	0.30 (0.26, 0.34)	0.51 (0.48, 0.58)	0.32 (0.30, 0.35)	0.53 (0.47, 0.61)	0.59 (0.46, 1.00)	0.43 (0.00, 0.56)	0.47 (0.28, 0.63)
Primaquine duration								
No primaquine	0 (0%)	0 (0%)	0 (0%)	0 (0%)	0 (0%)	196 (20%)	0 (0%)	196 (11%)
7 days	0 (0%)	0 (0%)	0 (0%)	0 (0%)	0 (0%)	385 (40%)	0 (0%)	385 (21%)
14 days	139 (100%)	331 (100%)	39 (100%)	51 (100%)	118 (100%)	382 (40%)	156 (100%)	1216 (68%)
Primaquine dose calculation								
No primaquine	0 (0%)	0 (0%)	0 (0%)	0 (0%)	0 (0%)	196 (20%)	0 (0%)	196 (11%)
Actual dosing	0 (0%)	0 (0%)	38 (97%)	0 (0%)	118 (100%)	767 (80%)	156 (100%)	1079 (60%)
Protocol dosing	139 (100%)	331 (100%)	1 (2.6%)	51 (100%)	0 (0%)	0 (0%)	0 (0%)	522 (29%)
Primaquine supervision								
No primaquine	0 (0%)	0 (0%)	0 (0%)	0 (0%)	0 (0%)	196 (20%)	0 (0%)	196 (11%)
Unsupervised	139 (100%)	0 (0%)	0 (0%)	0 (0%)	0 (0%)	0 (0%)	67 (43%)	206 (11%)
Partially supervised	0 (0%)	0 (0%)	0 (0%)	0 (0%)	0 (0%)	0 (0%)	89 (57%)	89 (5.0%)
Fully supervised	0 (0%)	331 (100%)	39 (100%)	51 (100%)	118 (100%)	767 (80%)	0 (0%)	1306 (73%)
Baseline haemoglobin level (g/dL)	11.0 (8.8, 12.4)	11.9 (10.8, 12.8)	14.3 (13.1, 14.7)	10.3 (9.5, 11.2)	13.8 (13.0, 14.5)	12.6 (11.4, 13.9)	11.6 (10.4, 13.1)	12.3 (11.0, 13.7)
Number of missing data	2	0	0	0	0	0	0	2
Day 7 methaemoglobin level (%)	NA	3.8 (2.7, 5.7)	5.6 (4.1, 7.1)	NA	5.8 (3.7, 8.2)	8.3 (5.4, 12)	NA	6.0 (3.5, 9.4)
Number of missing data	139	28	1	51	0	536	156	911
Transmission intensity								
Low	0 (0%)	0 (0%)	0 (0%)	0 (0%)	0 (0%)	0 (0%)	0 (0%)	0 (0%)
Moderate	0 (0%)	331 (100%)	0 (0%)	0 (0%)	0 (0%)	963 (100%)	156 (100%)	1450 (81%)
High	139 (100%)	0 (0%)	39 (100%)	51 (100%)	118 (100%)	0 (0%)	0 (0%)	347 (19%)
Origin of infections								
Within Papua	139 (100%)	0 (0%)	39 (100%)	0 (0%)	118 (100%)	0 (0%)	156 (100%)	452 (25%)
Outside Papua	0 (0%)	331 (100%)	0 (0%)	51 (100%)	0 (0%)	963 (100%)	0 (0%)	1345 (75%)
Study duration								
At least 180 days	0 (0%)	331 (100%)	39 (100%)	0 (0%)	118 (100%)	963 (100%)	156 (100%)	1607 (89%)
Less than 180 days	139 (100%)	0 (0%)	0 (0%)	51 (100%)	0 (0%)	0 (0%)	0 (0%)	190 (11%)

Numbers are in median (first quartile [Q1], third quartile [Q3]) or frequency (percentage). NA, not available; kg, kilogramme; μL, microlitre; g, gram; dL, decilitre; mg, milligramme. Transmission intensity was categorised as low (<1 case per 1000 person-years), moderate (1 case to <10 cases per 1000 person-years), and high (≥ 10 cases per 1000 person-years) according to subnational malaria incidence estimates for the median year of study enrolment.^29^ Patients contributing to the tolerability and safety datasets, as well as any sensitivity or restricted analyses, were subsets of the dataset shown in this table.

**Fig. 2:**
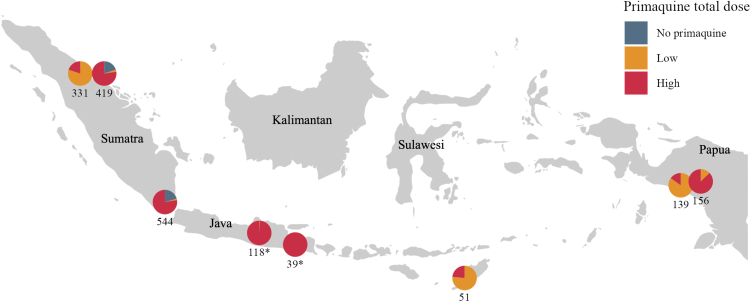
**Study sites that contributed to the pooled individual patient data across Indonesia.** Each circle represents a study site, and the number of participants included in the pooled data. Primaquine total dose: no primaquine (0 mg/kg), low (≥2 and < 5 mg/kg), high (≥5 mg/kg). Basemap shapefile data were obtained from the publicly available Natural Earth project (https://www.naturalearthdata.com) and accessed via the open-source R packages maps^24^ and ggplot2.^25^ ∗ Malaria-naive soldier patients were deployed and acquired infections in Papua then returned to malaria-free Java for follow-up (also referred to as the soldier relapse model^11^^,^^13^). For a more detailed description of the study sites, please refer to Table S4.^26^

The median age of the patients was 17 years (interquartile range [IQR] 10–29) with 172 (9.6%) younger than 5 years. The median weight was 45 kg (IQR 24–57) and 1056 (59%) were male. Overall, 452 (25%) patients acquired their *P. vivax* infection in Papua (Fig. 2) and 1432 (80%) were treated with dihydroartemisinin-piperaquine (Table 1). A total of 196 (11%) patients were treated without primaquine, 464 (26%) with low total dose, and 1137 (63%) with high total dose (Figures S5, S6, S7 and S8).

Compared with low total dose, patients administered high-dose primaquine were more likely to be treated over seven days, receive dihydroartemisinin-piperaquine, have a higher baseline parasite density, and have actual dosing data available (Table S3, Figure S9). Eligible studies that were not included^30^, ^31^, ^32^ predominantly enrolled patients in Papua and had a shorter follow-up duration (Tables S4, S5, and S6).

**Fig. 3:**
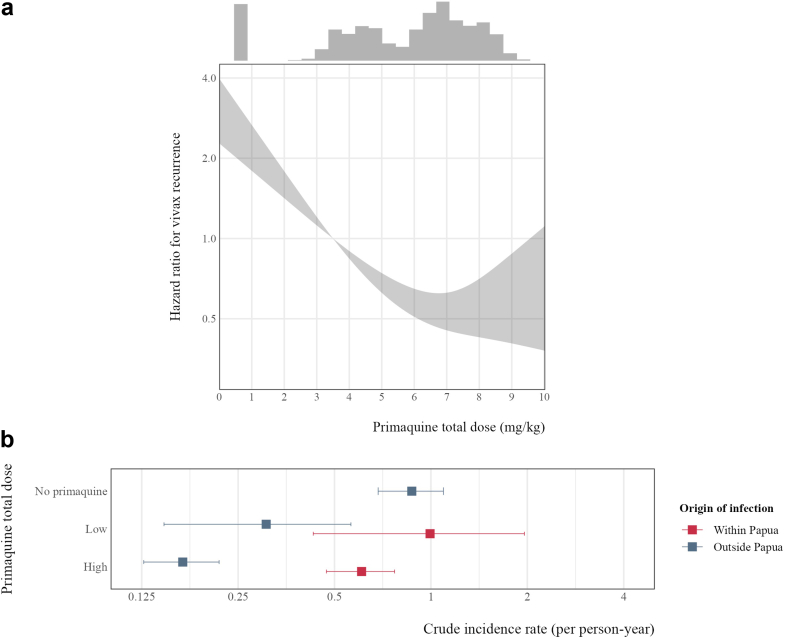
**Effect of increasing primaquine total dose on the rate of *P. vivax* recurrence by day 180.** (A) The reference value (HR = 1) was set at the target low dose of 3.5 mg/kg. The shaded region shows 95% CI. Estimates were derived from a multivariable Cox proportional hazards model, fitted to the primary efficacy dataset (n = 1797). The histogram along the top margin shows the distribution of primaquine total doses, with the leftmost bar representing patients who were treated without primaquine (i.e., 0 mg/kg). The vertical axis is shown on a logarithmic scale. (B) Crude incidence rates in the subset of patients (n = 1607) followed for up to 180 days across multiple *P. vivax* episodes by primaquine total dose and origin of infection. Patients treated without primaquine were only available from outside Papua. Point estimates (solid squares) were calculated as the number of *P. vivax* episodes divided by person-years at risk, with 95% CI for a Poisson rate obtained using an exact method. The horizontal axis is shown on a logarithmic scale. For a more detailed description of the study sites and the regression output, please refer to Tables S4 and S7, respectively.

Compared with patients treated without primaquine (enrolled in a single study conducted at non-Papuan sites), the rate of *P. vivax* recurrence by day 180 was lower in both patients treated with low-dose (3.5 mg/kg) primaquine (adjusted hazard ratio [AHR] 0.33; 95% CI 0.25–0.44) and high-dose (7 mg/kg) primaquine (AHR 0.18; 95% CI 0.12–0.26). The corresponding rate of recurrence was lower following high-dose primaquine compared to low-dose primaquine (AHR 0.53; 95% CI 0.45–0.63; Fig. 3). Increasing the total dose above 7 mg/kg remains of uncertain efficacy as the interval estimates of the estimated dose–response curve were wide (Fig. 3). This dose-response relationship was consistent across alternative dose groupings and sensitivity analyses (Figures S11 and S12). Negative control methods suggest residual confounding was minimal (List S3). The effect of increasing the dose of primaquine did not differ with age (<5 or ≥5 years old, p_interaction_ = 0.88) or by origin of infection (within Papua or outside Papua, p_interaction_ = 0.45, Figure S13).

In a subset of patients (n = 1608, 89%) followed through multiple *P. vivax* episodes for at least 180 days, those who acquired infections within Papua had a higher 180-day recurrence rate (76 episodes per 120 person-years; crude incidence rate [IR] 0.63 per person-year; 95% confidence interval [CI] 0.50–0.79) than those infected outside Papua (140 episodes per 451 person-years; IR 0.31 per person-year; 95% CI 0.26–0.37). Fig. 3 shows recurrence rates for both regions by primaquine dose groupings.

Gastrointestinal tolerability on days 5–7 was assessed in 1254 (70%) patients from five studies.^12^^,^^14^^,^^15^^,^^27^^,^^28^ Of these, 190 (15%) received no primaquine, 203 (16%) received low daily dose, 482 (38%) intermediate daily dose, and 379 (30%) high daily dose primaquine; with 125 (10%) younger than 5 years. Within each primaquine daily dose group, there was substantial heterogeneity in the percentage of patients reporting gastrointestinal discomfort on days 5–7 across studies (Figure S14, Table S7), making data pooling for effect estimation unreliable. To estimate the unbiased effect of increasing daily doses, we used subsets of data from one trial^15^ in which primaquine daily doses were randomised. The risk of early gastrointestinal symptoms (day 0 and days 1–2) did not vary with the daily dose of primaquine (Figure S15, Table S9). However, by days 5–7, when 894/952 (94%) patients were afebrile, the risk of gastrointestinal symptoms increased with the daily dose of primaquine: adjusted risk ratio [ARR] 1.32 (95% CI 1.15–1.51) per 0.25 mg/kg/day increment in primaquine dose (Figure S15, Table S9). In 767 patients assessable for acute vomiting, there was inconclusive evidence that a higher daily dose of primaquine resulted in an increased risk of acute vomiting within 60 min of administration (ARR 1.19; 95% CI 0.84–1.67 per 0.25 mg/kg/day increment; Figure S16, Table S8).

One multi-country randomised trial^15^ contributed 822 patients to the primary haematological safety analysis, of whom 173 (21%) received no primaquine, 6 (<1%) received a low daily dose, 318 (39%) received an intermediate daily dose, and 325 (40%) a high daily dose. G6PD activity was measured at baseline in all eligible patients and was similar between treatment arms; 788 (96%) had normal G6PD activity (≥70%) and 34 (4%) had intermediate activity (30–69%; Figure S17).

Severe haemolysis was recorded in one patient, a female in the 5–14-year age group with 37.4% G6PD activity treated with dihydroartemisinin-piperaquine plus a high daily dose of primaquine (0.99 mg base/kg) administered over 7 days. She had a 41% fall in haemoglobin from 11.6 g/dL on day 0–6.9 g/dL on day 3 (4.7 g/dL reduction) and recovered (Hb ≥ 11 g/dL) at least by day 21 without requiring a blood transfusion. No patients had a haemoglobin fall to <5 g/dL, a haemoglobin fall >5 g/dL from baseline, blood transfusion, renal failure requiring dialysis, or death (Table S10).

**Fig. 4:**
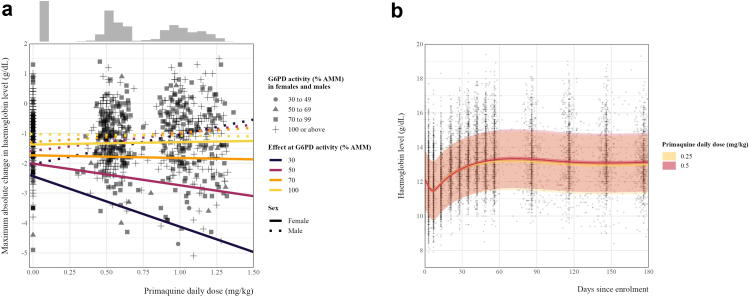
**Effect of primaquine daily dose on haematological profile.** (A) Maximum absolute change from baseline in haemoglobin levels between days 1 and 14 across primaquine daily doses, sex, and G6PD activity levels. Estimates were derived from a multivariable linear model fitted to the primary safety dataset (n = 822, 34 patients with intermediate G6PD activity between 30% and 69%). The plot shows model-implied predictions at four selected cut-offs (30%, 50%, 70%, and 100%) of G6PD activity to aid interpretation. The histogram along the top margin shows the distribution of primaquine daily doses in the model data, with the leftmost bar representing patients who were treated without primaquine (i.e., 0 mg/kg). (B) Temporal dynamics of haematological recovery in patients with G6PD activity of at least 70%. Estimates were derived from a multilevel, multivariable linear model (n = 790). Model-implied predictions at primaquine daily doses of 0.25 mg/kg and 0.5 mg/kg are shown. Solid curves represent the expected haemoglobin levels over time following treatment, with thick and thin shaded regions denoting the 95% confidence and prediction intervals, respectively. AMM, adjusted male median. For a more detailed description of the regression output, please refer to Table S7.

The effect of primaquine daily dose on haemoglobin varied with sex and G6PD activity (p_interaction_ = 0.01). In contrast to females with normal G6PD activity, a higher daily dose of primaquine was associated with a greater absolute fall in haemoglobin in females with intermediate G6PD activity (Fig. 4A, Figure S18). In males, lower G6PD activity was not associated with a greater absolute decrease in haemoglobin. In 612 patients with normal G6PD activity and a baseline haemoglobin level of ≥11 g/dL, the risk of anaemia on days 2–3 did not increase with higher primaquine daily doses (Figure S19, Table S9). In 788 patients with normal G6PD activity, haemoglobin levels reached an expected nadir on days 2–3, likely reflecting haemolysis associated with blood-stage acute malaria. Haemoglobin levels returned to baseline approximately two weeks after initiating treatment (Fig. 4B). A higher primaquine daily dose resulted in higher day 7 methaemoglobin levels and an increased risk of methaemoglobin levels ≥10% between days 1–14 (prevalence = 262/1026 [25.5%], Figure S20).

## Discussion

The optimal anti-relapse regimen to prevent recurrent *P. vivax* malaria must trade-off the absolute number of recurrent malaria episodes averted against the length of treatment duration, its tolerability, and safety. This balance of practicality, tolerability, and safety against absolute benefit will vary across regions primarily because the absolute risk of recurrence varies considerably. Our meta-analysis of 1797 Indonesian patients demonstrates that increasing the total dose of primaquine from 3.5–7 mg/kg would halve the recurrence rate. However, the absolute benefit provided by the higher dose will vary substantially as the crude recurrence incidence rate varied substantially between regions (IR 0.63 [95% CI 0.50–.79] per person-year within Papua; IR 0.31 [95% CI 0.26–0.37] per person-year outside Papua). Higher daily doses were associated with a greater risk of gastrointestinal discomfort and there is a risk of severe primaquine-induced haemolysis in females with intermediate G6PD activity.^33^ Identifying an optimal balance between benefits and harms is key to determining the best regimen for each region within Indonesia.

**Fig. 5:**
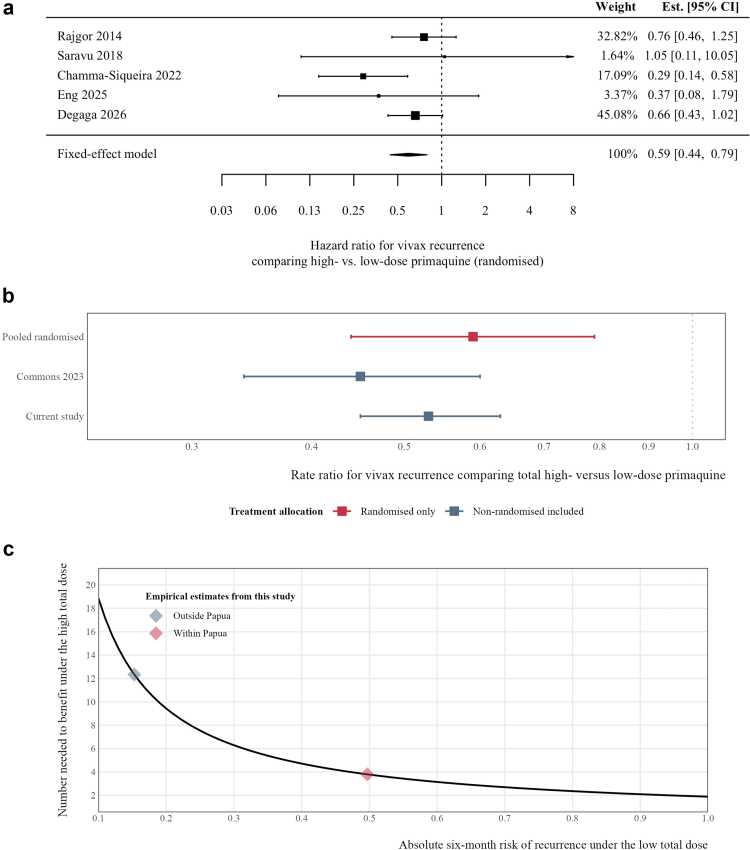
**Meta-analysis of randomised controlled trials and its comparison to global and Indonesian IPD meta-analyses on the effect of high-dose versus low-dose primaquine.** (A) Systematic search for trials that randomised high- and low-dose primaquine was conducted without country restrictions from 1960 to 2025 with a minimum follow-up of approximately six months. Estimates from the first two studies^35^^,^^36^ were derived from the number of events and the period at risk reported in each study to calculate the incidence rate ratio (patients censored before the end of the study were assumed to contribute half of their respective follow-up period). Estimates from the last three studies^37^, ^38^, ^39^ were obtained directly from the hazard ratio reported in each study. In the current context, these effect measures (i.e., the incidence rate ratio and hazard ratio) were assumed to be approximately interchangeable and summarised here as the rate ratio. Vertical dashed line indicates an equal effect (RR = 1). Estimates to the left of the dashed line indicate greater efficacy of high dose primaquine. Estimates to the right indicate greater efficacy of low dose primaquine. (B) Pooled randomised estimates were derived from a study-level global meta-analysis presented in the top panel. Estimates from Commons 2023^6^ were derived from an IPD meta-analysis of randomised and non-randomised *P. vivax* efficacy studies conducted globally. Estimates from the current study were derived by comparing the hazard ratio estimate at 7 mg/kg with that at 3.5 mg/kg in the Indonesian pooled data. The solid square represents a point estimate with a 95% confidence interval. The horizontal axis is shown on a logarithmic scale. CI, confidence interval. IPD, individual patient data. (C) Expected number needed to benefit with total high-dose primaquine (compared with total low-dose primaquine, assuming a risk ratio of 0.53) across varying absolute risks of recurrence over a 6-month period.

Our Indonesia-specific estimates for anti-relapse efficacy comparing high- and low-dose primaquine align with those reported by Commons and colleagues using pooled global data.^6^ Both analyses included non-randomised comparisons, so confounding bias cannot be excluded. One limitation of our study is that all patients included in the no-primaquine group were from sites outside Papua, and there was no direct randomisation to the low-dose group. Much of the within-study dose variation was attributable to differences in body weight rather than to random allocation. Similar to Commons et al.,^6^ we used a causal diagram to inform adjustment for key confounders in regression analysis. We further applied negative control methods^34^ and found no material residual confounding. Consistent with this study, a meta-analysis of study-level data from randomised trials^35^, ^36^, ^37^, ^38^, ^39^ directly comparing high- and low-dose primaquine in any country supported that high-dose primaquine reduces the rate of first *P. vivax* recurrence by around 50% (Fig. 5A and B).

With intense, year-round malaria transmission, infections in Papua may involve a higher hypnozoite burden, as well as, ‘Chesson-like’ strains that are more tolerant to primaquine, both necessitating a higher primaquine dose.^40^, ^41^, ^42^ In contrast, regions outside Papua, such as Sumatra, experience low to moderate and increasingly focal transmission approaching elimination, where residual burden is often linked to mobile and migrant populations and *P. vivax* predominance.^43^ Lower force of reinfection means observed recurrences may be more likely to reflect hypnozoite activation, and treatment responses could therefore appear more favourable than in Papua. However, we found no evidence that higher doses of primaquine in patients infected in Papua would provide greater relative efficacy compared to patients infected outside Papua. The global analysis reported similar findings, showing high relative efficacy of high-dose primaquine in locations with both low- and high-relapse periodicity.^6^^,^^26^ Hence, higher relative anti-relapse efficacy of increased primaquine doses is likely consistent and generalisable across different regions and parasite strains. However, given the diverse case-mix and varying baseline risks across populations, heterogeneous treatment-effects on an absolute scale are expected and will be relevant for policy makers and clinicians. When comparing high versus low total-dose primaquine, the number needed to benefit to prevent one recurrence over six months in Papua would be around four patients, compared with about twelve patients in non-Papua regions (Fig. 5C).

A higher daily dose of primaquine led to increased gastrointestinal discomfort, independent of acute malaria symptoms. Reduced tolerability could lead to non-compliance, preventing the target total dose from being achieved. Reduced adherence or inadequate supervision may lead to lower anti-relapse efficacy.^12^^,^^44^ Although, previous studies have shown that taking primaquine tablets with a meal significantly improves gastrointestinal tolerability,^45^ relevant data to address this in our IPD analysis were not available.^46^

A lack of routine G6PD screening limits the safe implementation of high total dose primaquine. The risk of haemolysis is almost entirely in individuals with G6PD deficiency. While our analysis did not include this population, our analysis included patients with intermediate G6PD activity. Chu and colleagues reported that despite being labelled as phenotypically normal by point-of-care tests (≥30% G6PD activity), G6PD heterozygous females can experience significant haemolysis when exposed to 1 mg/kg/day of primaquine.^33^ We observed that females with G6PD activity between 30 and 69% experienced greater haemoglobin reductions as the daily dose increased compared to those with G6PD activity ≥70%, however this was not clinically significant at doses of 0.5 mg/kg/day or less. Primaquine also induced dose-dependent increases in methaemoglobin levels in our study population. Prior evidence suggests that this elevation typically occurs without symptoms, and resolves spontaneously within weeks.^45^^,^^47^ Increased primaquine-induced methaemoglobinaemia indicates sufficient production of active primaquine metabolites and hence, greater anti-relapse efficacy.^48^^,^^49^

In line with the 2024 WHO antimalarial guidelines,^8^ our findings suggest that for most patients with *P. vivax* in Indonesia, an optimal regimen could involve a high total dose of 7 mg/kg administered over 1–2 weeks, taken with food, and initiated after screening for G6PD deficiency. Of note, in western Indonesia, there is a low risk of relapse following a low total dose primaquine,^14^ suggesting that the absolute benefit from high total dose primaquine may be reduced in these areas and different dosing could be considered. A pragmatic next step could be a tiered national strategy aligned to administrative boundaries, with 7 mg/kg prioritised in higher-relapse settings and lower doses considered in lower-risk areas, supported by robust surveillance of recurrence and safety outcomes. Mathematical scenario modelling could further inform optimal doses under varying local transmission and programmatic conditions.

Tafenoquine has been recommended for use in endemic countries in South America, where chloroquine is typically the blood-stage drug of choice.^8^ This single-dose anti-relapse treatment is currently not recommended for patients receiving artemisinin-based combination therapies (ACTs).^8^ In Indonesia, where ACTs are recommended due to a high prevalence of chloroquine-resistant *P. vivax*, tafenoquine is not currently available. The INSPECTOR trial, conducted in soldiers returning from Indonesian Papua, found no clinically meaningful benefit of tafenoquine plus dihydroartemisinin-piperaquine compared with dihydroartemisinin-piperaquine alone.^30^ The reason for this remains unclear, although a drug–drug interaction or suboptimal dosing have been proposed.^50^ A randomised pharmacokinetic trial found no evidence of reductions in tafenoquine exposure when co-administered with dihydroartemisinin-piperaquine.^51^ Ongoing trials (e.g., TADORE+ [NCT07060794], SEADOT [NCT04704999]) will further assess potential drug–drug interactions and whether suboptimal dosing may account for the observed discrepancies.

We were unable to include two recently published eligible studies in our pooled dataset, primarily due to unavailability of IPD. These studies would have added <10% more patients, making it unlikely that the current results would change significantly. One of these studies^30^ randomised patients to receive low-dose primaquine over 14 days versus a placebo, and its estimated efficacy (HR 0.26; 95% CI 0.16–0.43) aligns with our pooled effect-estimates. In the current study, ∼30% of patients exposed to primaquine were estimated from the study protocol rather than the actual tablets administered. Given an inability to account for reduced adherence this may have led to the actual dose of primaquine received being less than expected, which would be expected to bias the anti-relapse efficacy towards the null, making our efficacy estimates conservative. While the same applies to tolerability and safety (i.e., higher daily doses potentially appearing more tolerable and safer), both in practice can be effectively mitigated by taking food and G6PD screening. Therefore, the impact of potential quantitative bias is deemed minimal for clinical use. Although our focus was on anti-relapse efficacy, the studies (except for those conducting follow-up in malaria-free regions^11^^,^^13^) were unable to distinguish whether recurrences were caused by new infections, treatment failure, or hypnozoite activation. The use of highly efficacious schizonticides made recrudescence unlikely and approximately 80% of vivax recurrences were estimated to result from relapses.^52^ If new infections were indeed substantial, they would have diluted our efficacy estimates towards the null, making our results conservative. Lastly, while underlying transmission intensity could confound the estimated causal effect and may vary across locations and over time, transmission levels were broadly stable within sites across the years. We therefore adjusted for study site to account for spatial and temporal differences, and a negative outcome control analysis using *P. falciparum* infection suggested that any residual confounding was minimal.

In summary, increasing the total primaquine dose from 3.5 mg/kg to 7 mg/kg is likely to provide a substantial reduction in the risk of recurrence, with no evidence of treatment-effect heterogeneity across Indonesia. However, the variable risk of recurrence across the country means that there are substantial differences in the absolute benefit of 7 mg/kg versus 3.5 mg/kg total dose primaquine (e.g., higher benefit in Papua but lower in Sumatra). Completing a high total dose of primaquine in a shorter timeframe by administering a higher daily dose, is preferable for maximising adherence to a full course of treatment, however this may increase the risk of gastrointestinal discomfort or haemolysis. These risks can be mitigated by co-administering primaquine with food, G6PD screening, and monitoring for potential haemolytic events. Future efforts should prioritise optimising implementation, adherence, and safe delivery of the regimens rather than pursuing evaluation of higher primaquine total doses above 7 mg/kg.

## Contributors

IF, JAW, RNP, JKB, and RJC conceived the study, analysed and interpreted the data, and drafted the manuscript. IF and RJC accessed and verified the data. JAW, RNP, and JKB provided technical support. APP, IS, EJN, KL, IRFE, WRJT, KT, NPJD, JRP, JAS, RNP, JKB conceived and undertook the individual studies and enrolled the patients. All authors revised the manuscript and were responsible for the decision to submit for publication.

## Data sharing statement

Pseudo-anonymised participant data used in this analysis are available for access via the Infectious Diseases Data Observatory (IDDO) website (https://www.iddo.org). Requests for data access will be reviewed according to IDDO procedures, to ensure that use of data protects the interests of the participants and researchers according to the terms of ethics approval and principles of equitable data sharing. IDDO is registered with the Registry of Research Data Repositories (https://www.re3data.org/). Code for data analysis and visualisation is available at https://github.com/ihsanfadil/wwarn-pq-ina.

## Declaration of generative AI and AI-assisted technologies in the manuscript preparation process

During the preparation of this work the author(s) used GPT-5 to perform spell-checking and review computer code for errors. After using this tool/service, the author(s) reviewed and edited the content as needed and take(s) full responsibility for the content of the published article.

## Editor note

The Lancet Group takes a neutral position with respect to territorial claims in published maps and institutional affiliations.

## Declaration of interests

JKB and KT report institutional research funding from Medicines for Malaria Venture. JKB also reports institutional research funding from GSK, Wellcome Trust, and Sanaria; participation on the US national Institutes of Health data safety monitoring board; and membership of the editorial board of *Travel Medicine and Infectious Disease* and the guidelines development group for malaria control and elimination, Global Malaria Programme, WHO. RJC has been a technical advisor for the WHO guidelines development group. RJC, JKB, and RNP report contributions to Up-to-Date. All other authors declare no competing interests.
