# Supplementary material for: Effect of higher dose primaquine for the radical cure of Plasmodium vivax malaria in Indonesia: a systematic review and individual patient data meta-analysis
Source: Lancet Reg Health West Pac. 2026 Jun 18;72:101908. doi: 10.1016/j.lanwpc.2026.101908 (PMC13310647; doi:10.1016/j.lanwpc.2026.101908)
Supplement: Supplementary Material [file mmc1.docx]

# Supplementary Material

List S1. Systematic search terms for the databases 2

List S2. Supplementary methods 3

List S3. Negative control results 26

Table S1. PRISMA-IPD checklist 5

Table S2. Risk of bias assessment 10

Table S3. Patient characteristics by primaquine total dose group 15

Table S4. Studies included in analysis 16

Table S5. Studies that were eligible for analysis but not included for data pooling 17

Table S6. Comparison of patient characteristics who received primaquine between included studies and eligible but not available studies 18

Table S7. Model estimates underlying the main results 22

Table S8. Pooled counts and percentages of patients who experienced gastrointestinal discomfort 28

Table S9. Pooled counts and percentages of patients who experienced acute vomiting within 1 hour of taking primaquine between days 0 and 14 30

Table S10. Poisson model estimates with robust standard errors 30

Table S11. Haematological adverse events on days 1–14, by dose group 31

Figure S1. Causal directed acyclic graph showing the relationship between the two factors of interest (red nodes): primaquine mg/kg total dose and the risk of P. vivax relapse 11

Figure S2. Causal directed acyclic graphs illustrating the use of P. falciparum infection and body weight as negative controls to detect residual confounding 12

Figure S3. Causal directed acyclic graph showing the relationship between the two factors of interest (red nodes): primaquine mg/kg daily dose and the risk of gastrointestinal (GI) discomfort 13

Figure S4. Causal directed acyclic graph showing the relationship between the two factors of interest (red nodes): primaquine mg/kg daily dose and haemolysis 13

Figure S5. Distribution of primaquine total dose by primaquine duration 14

Figure S6. Distribution of primaquine daily dose by primaquine regimen 14

Figure S7. Primaquine total dose received across different patient age 19

Figure S8. Primaquine total dose by schizontocidal drug (A) and study (B) 19

Figure S9. Primaquine total dose by body weight in patients receiving primaquine 20

Figure S10. Distributions of the days to first P. vivax recurrence by study 21

Figure S11. P. vivax recurrence at day 180 by different groups 23

Figure S12. Sensitivity analyses of the hazard ratio across primaquine total dose 25

Figure S13. Estimated hazard ratios of P. vivax recurrence at day 180, assuming effect modification by (A) origin of infection and (B) age category 27

Figure S14. Heterogeneity in percentages of gastrointestinal discomfort on days 5–7 by study and primaquine daily dose 28

Figure S15. Risk ratio of gastrointestinal discomfort (A) between days 5 to 7, (B) between days 1 to 2, and (C) at day 0 [baseline] 29

Figure S16. Risk ratio of acute vomiting within 1 hour of taking primaquine between days 0 and 14 30

Figure S17. (A) Haemoglobin concentrations and (B) G6PD activity levels at patient enrolment in the primary safety dataset (n = 822) 31

Figure S18. Maximum absolute change from baseline in haemoglobin levels (A) between days 2 and 3 and (B) between days 5 and 7 across primaquine daily doses, sex, and G6PD activity levels 32

Figure S19. Risk ratio of developing anaemia (haemoglobin <11 g/dL) between days 2 to 3 in patients with G6PD activity of at least 70% and had baseline haemoglobin level of at least 11 g/dL 33

Figure S20. Effect of primaquine daily dose on methaemoglobin levels 34

**List S1. Systematic search terms for the databases**

Vivax AND (artefenomel OR arterolane OR amodiaquine OR atovaquone OR artemisinin OR arteether OR artesunate OR artemether OR artemotil OR azithromycin OR artekin OR chloroquine OR chlorproguanil OR cycloguanil OR clindamycin OR coartem OR dapsone OR dihydroartemisinin OR duo-cotecxin OR doxycycline OR halofantrine OR lumefantrine OR lariam OR malarone OR mefloquine OR naphthoquine OR naphthoquinone OR piperaquine OR primaquine OR proguanil OR pyrimethamine OR pyronaridine OR proguanil OR quinidine OR quinine OR riamet OR sulphadoxine OR tetracycline OR tafenoquine)

**List S2. Supplementary methods**

**Additional inclusion criteria**

For the haematological safety analysis, only patients with G6PD activity ≥30% of the adjusted male median (AMM) or a negative qualitative test were included. Reporting of parasite presence or absence during follow-up, presence of gastrointestinal discomfort (vomiting, anorexia, diarrhoea) during follow-up, and haemoglobin measured on day 0 and at least once during follow up were required for anti-relapse efficacy, gastrointestinal tolerability, and haematological safety analyses, respectively. For Poespoprodjo 2022,^12^ we included only patients for whom actual dose data were available. Protocol-based dose calculation was considered unreliable in the context of unsupervised primaquine.

**Categories of total and daily primaquine doses**

| **Category** | **Body weight-adjusted primaquine exposure (mg/kg)** | |
| --- | --- | --- |
|  | **Daily** | **Total** |
| No primaquine | 0 | 0 |
| Very low | ··· | >0 and <2 |
| Low | >0 and <0·375 | ≥2 and <5 |
| Intermediate | ≥0·375 and <0·75 | ··· |
| High | ≥0·75 | ≥5 |

**Follow-up periods of the primary endpoints**

| **Analysis** | **Follow-up period** | **Rationale** |
| --- | --- | --- |
| Anti-relapse efficacy | 7–180 days | For patients followed in endemic settings, the cause of *P. vivax* recurrence cannot be determined with certainty as recrudescence, relapse, or reinfection. Early recurrences are more likely to represent recrudescence; therefore, events occurring within the first week were excluded. In addition, the proportion of recurrences attributable to relapse is expected to decline over time as reinfections become more common with increasing exposure. The 7–180 day window was selected as a pragmatic balance to primarily capture recurrences likely due to hypnozoite activation (i.e., relapse). |
| Gastrointestinal tolerability | 5–7 days | Acute malaria symptoms and blood-stage treatment can themselves cause gastrointestinal disturbances similar to those resulting from primaquine. We therefore selected days 5–7, when primaquine administration was ongoing but schizontocidal treatment had been completed and acute malaria symptoms had largely resolved. This window was chosen to better attribute gastrointestinal symptoms to primaquine exposure. |
| Haematological safety | 1–14 days | The highest risk of primaquine-induced haemolysis occurs during the first few days after treatment initiation while the drug is being administered. The first two or three days were expected to capture the most clinically relevant early haemoglobin declines. The 1–14 day window was selected to encompass the full duration of treatment, as regimens varied between 7 and 14 days. |

**Data analysis: anti-relapse efficacy**

Variables violating proportional hazards assumptions were adjusted accordingly. Patients were censored at recurrence, new antimalarial treatment, loss to follow-up, study end, outcome assessment, or a smear gap >67 days.

To detect residual confounding, we used negative control methods,^30^ using *P. falciparum* recurrence and patient body weight (see **Figure S*2*** for the rationale). Sensitivity analyses restricted the event of interest to symptomatic *P. vivax* recurrent parasitaemia and excluded unsupervised regimens, as well as data from the sole cluster-randomised trial. In studies with ≥180 days follow-up that tracked patients through multiple episodes of *P. vivax* parasitaemia, the incidence of recurrence was also analysed using Poisson regression. Rates were calculated as episodes per person-year, with censoring consistent with the Cox model.

**Data analysis: haematological safety**

The analysis of absolute fall in haemoglobin level was repeated for endpoints on days 2–3 and 5–7. Among patients with normal G6PD activity and baseline haemoglobin ≥11 g/dL, risk of anaemia by days 2–3 was modelled with Poisson regression using the same covariates, excluding G6PD level. Haematological recovery in this G6PD group was modelled with a multilevel linear model using time, daily primaquine dose, baseline haemoglobin, age, sex, log-transformed baseline parasite density, study site, and patient-specific intercepts. Splines and interaction terms were used for time and dose. The effect of daily primaquine dose on the day 7 methaemoglobin concentration and the risk of clinical methaemoglobinaemia (i.e., 10% or above) between days 1 and 14 was modelled using multivariable linear and Poisson regression, respectively; including age (years), sex (male or female), baseline parasite density (parasites per μL of blood; natural log transformed), and study site as covariates. We fitted this model by incorporating a nonlinear effect using a restricted cubic spline.

**Table S1. PRISMA-IPD checklist**

| **PRISMA-IPD**  **Section/topic** | **Item No** | **Checklist item** | **Reported on page** |
| --- | --- | --- | --- |
| Title | | | |
| Title | 1 | Identify the report as a systematic review and meta-analysis of individual participant data. | 1 |
| **Abstract** | | | |
| Structured summary | 2 | Provide a structured summary including as applicable: | 2 |
|  |  | Background: state research question and main objectives, with information on participants, interventions, comparators and outcomes. |  |
|  |  | Methods: report eligibility criteria; data sources including dates of last bibliographic search or elicitation, noting that IPD were sought; methods of assessing risk of bias. |  |
|  |  | Results: provide number and type of studies and participants identified and number (%) obtained; summary effect estimates for main outcomes (benefits and harms) with confidence intervals and measures of statistical heterogeneity. Describe the direction and size of summary effects in terms meaningful to those who would put findings into practice. |  |
|  |  | Discussion: state main strengths and limitations of the evidence, general interpretation of the results and any important implications. |  |
|  |  | Other: report primary funding source, registration number and registry name for the systematic review and IPD meta-analysis. |  |
| **Introduction** | | | |
| Rationale | 3 | Describe the rationale for the review in the context of what is already known. | 4 |
| Objectives | 4 | Provide an explicit statement of the questions being addressed with reference, as applicable, to participants, interventions, comparisons, outcomes and study design (PICOS). Include any hypotheses that relate to particular types of participant-level subgroups. | 4 |
| **Methods** | | | |
| Protocol and registration | 5 | Indicate if a protocol exists and where it can be accessed. If available, provide registration information including registration number and registry name. Provide publication details, if applicable. | 5 |
| Eligibility criteria | 6 | Specify inclusion and exclusion criteria including those relating to participants, interventions, comparisons, outcomes, study design and characteristics (e.g. years when conducted, required minimum follow-up). Note whether these were applied at the study or individual level i.e. whether eligible participants were included (and ineligible participants excluded) from a study that included a wider population than specified by the review inclusion criteria. The rationale for criteria should be stated. | 5 |
| Identifying studies - information sources | 7 | Describe all methods of identifying published and unpublished studies including, as applicable: which bibliographic databases were searched with dates of coverage; details of any hand searching including of conference proceedings; use of study registers and agency or company databases; contact with the original research team and experts in the field; open adverts and surveys. Give the date of last search or elicitation. | 5 |
| Identifying studies - search | 8 | Present the full electronic search strategy for at least one database, including any limits used, such that it could be repeated. | 5 |
| Study selection processes | 9 | State the process for determining which studies were eligible for inclusion. | 5 |
| Data collection processes | 10 | Describe how IPD were requested, collected and managed, including any processes for querying and confirming data with investigators. If IPD were not sought from any eligible study, the reason for this should be stated (for each such study). | 5 |
|  |  | If applicable, describe how any studies for which IPD were not available were dealt with. This should include whether, how and what aggregate data were sought or extracted from study reports and publications (such as extracting data independently in duplicate) and any processes for obtaining and confirming these data with investigators. |  |
| Data items | 11 | Describe how the information and variables to be collected were chosen. List and define all study level and participant level data that were sought, including baseline and follow-up information. If applicable, describe methods of standardising or translating variables within the IPD datasets to ensure common scales or measurements across studies. | 5 |
| IPD integrity | A1 | Describe what aspects of IPD were subject to data checking (such as sequence generation, data consistency and completeness, baseline imbalance) and how this was done. | 5 |
| Risk of bias assessment in individual studies. | 12 | Describe methods used to assess risk of bias in the individual studies and whether this was applied separately for each outcome. If applicable, describe how findings of IPD checking were used to inform the assessment. Report if and how risk of bias assessment was used in any data synthesis. | 7 |
| Specification of outcomes and effect measures | 13 | State all treatment comparisons of interests. State all outcomes addressed and define them in detail. State whether they were pre-specified for the review and, if applicable, whether they were primary/main or secondary/additional outcomes. Give the principal measures of effect (such as risk ratio, hazard ratio, difference in means) used for each outcome. | 5–6 |
| Synthesis methods | 14 | Describe the meta-analysis methods used to synthesise IPD. Specify any statistical methods and models used. Issues should include (but are not restricted to):   - Use of a one-stage or two-stage approach. - How effect estimates were generated separately within each study and combined across studies (where applicable). - Specification of one-stage models (where applicable) including how clustering of patients within studies was accounted for. - Use of fixed or random effects models and any other model assumptions, such as proportional hazards. - How (summary) survival curves were generated (where applicable). - Methods for quantifying statistical heterogeneity (such as I^2^ and t^2^). - How studies providing IPD and not providing IPD were analysed together (where applicable). - How missing data within the IPD were dealt with (where applicable). | 5–6 |
| Exploration of variation in effects | A2 | If applicable, describe any methods used to explore variation in effects by study or participant level characteristics (such as estimation of interactions between effect and covariates). State all participant-level characteristics that were analysed as potential effect modifiers, and whether these were pre-specified. | 5–6 |
| Risk of bias across studies | 15 | Specify any assessment of risk of bias relating to the accumulated body of evidence, including any pertaining to not obtaining IPD for particular studies, outcomes or other variables. | 8 |
| Additional analyses | 16 | Describe methods of any additional analyses, including sensitivity analyses. State which of these were pre-specified. | 5–6 |
| **Results** | | | |
| Study selection and IPD obtained | 17 | Give numbers of studies screened, assessed for eligibility, and included in the systematic review with reasons for exclusions at each stage. Indicate the number of studies and participants for which IPD were sought and for which IPD were obtained. For those studies where IPD were not available, give the numbers of studies and participants for which aggregate data were available. Report reasons for non-availability of IPD. Include a flow diagram. | 8 |
| Study characteristics | 18 | For each study, present information on key study and participant characteristics (such as description of interventions, numbers of participants, demographic data, unavailability of outcomes, funding source, and if applicable duration of follow-up). Provide (main) citations for each study. Where applicable, also report similar study characteristics for any studies not providing IPD. | 8 |
| IPD integrity | A3 | Report any important issues identified in checking IPD or state that there were none. | 8 |
| Risk of bias within studies | 19 | Present data on risk of bias assessments. If applicable, describe whether data checking led to the up-weighting or down-weighting of these assessments. Consider how any potential bias impacts on the robustness of meta-analysis conclusions. | 8 |
| Results of individual studies | 20 | For each comparison and for each main outcome (benefit or harm), for each individual study report the number of eligible participants for which data were obtained and show simple summary data for each intervention group (including, where applicable, the number of events), effect estimates and confidence intervals. These may be tabulated or included on a forest plot. | 8 |
| Results of syntheses | 21 | Present summary effects for each meta-analysis undertaken, including confidence intervals and measures of statistical heterogeneity. State whether the analysis was pre-specified, and report the numbers of studies and participants and, where applicable, the number of events on which it is based. | 8–9 |
|  |  | When exploring variation in effects due to patient or study characteristics, present summary interaction estimates for each characteristic examined, including confidence intervals and measures of statistical heterogeneity. State whether the analysis was pre-specified. State whether any interaction is consistent across trials. |  |
|  |  | Provide a description of the direction and size of effect in terms meaningful to those who would put findings into practice. |  |
| Risk of bias across studies | 22 | Present results of any assessment of risk of bias relating to the accumulated body of evidence, including any pertaining to the availability and representativeness of available studies, outcomes or other variables. | 8 |
| Additional analyses | 23 | Give results of any additional analyses (e.g. sensitivity analyses). If applicable, this should also include any analyses that incorporate aggregate data for studies that do not have IPD. If applicable, summarise the main meta-analysis results following the inclusion or exclusion of studies for which IPD were not available. | 8–9 |
| **Discussion** | | | |
| Summary of evidence | 24 | Summarise the main findings, including the strength of evidence for each main outcome. | 10 |
| Strengths and limitations | 25 | Discuss any important strengths and limitations of the evidence including the benefits of access to IPD and any limitations arising from IPD that were not available. | 10–12 |
| Conclusions | 26 | Provide a general interpretation of the findings in the context of other evidence. | 10–12 |
| Implications | A4 | Consider relevance to key groups (such as policy makers, service providers and service users). Consider implications for future research. | 10–12 |
| **Funding** | | | |
| Funding | 27 | Describe sources of funding and other support (such as supply of IPD), and the role in the systematic review of those providing such support. | 7 |

**Table S2. Risk of bias assessment**

| **Study** | **Analysis** | **Domain based on ROB2 for randomised trials** | | | | | | | | **Overall** |
| --- | --- | --- | --- | --- | --- | --- | --- | --- | --- | --- |
|  |  | **Randomisation** | **Intervention** | | **Outcome** | | **Reported results** | | |  |
|  |  |  |  |  | **Missingness** | **Measurement** |  |  |  |  |
| **Taylor 2019** | Efficacy | Low | Low | | Low | Low | ·· | | | Low |
|  | Tolerability | Low | Low | | Low | Low | ·· | | | Low |
|  | Safety | Low | Low | | Low | Low | ·· | | | Low |
|  |  | | |  | | | | | | |
| **Study** | **Analysis** | **Domain based on ROBINS-I for non-randomised studies of interventions** | | | | | | | | **Overall** |
|  |  | **Confounding** | **Intervention classification** | | **Selection** | **Intervention deviation** | **Missingness** | **Outcome**  **measurement** | **Reported results** |  |
| **Hasugian 2007** | Efficacy | Low | Moderate | | Low | Low | Low | Low | ·· | Moderate |
|  | Tolerability | Low | Moderate | | Low | Low | Moderate | Low | ·· | Moderate |
|  | Safety | ·· | ·· | | ·· | ·· | ·· | ·· | ·· | ·· |
| **Pasaribu 2013** | Efficacy | Low | Moderate | | Low | Low | Low | Low | ·· | Moderate |
|  | Tolerability | Low | Moderate | | Low | Low | Moderate | Low | ·· | Moderate |
|  | Safety | ·· | ·· | | ·· | ·· | ·· | ·· | ·· | ·· |
| **Sutanto 2013** | Efficacy | Low | Low | | Low | Low | Low | Low | ·· | Low |
|  | Tolerability | ·· | ·· | | ·· | ·· | ·· | ·· | ·· | ·· |
|  | Safety | ·· | ·· | | ·· | ·· | ·· | ·· | ·· | ·· |
| **Lidia 2015** | Efficacy | Low | Moderate | | Low | Low | Low | Low | ·· | Moderate |
|  | Tolerability | ·· | ·· | | ·· | ·· | ·· | ·· | ·· | ·· |
|  | Safety | ·· | ·· | | ·· | ·· | ·· | ·· | ·· | ·· |
| **Nelwan 2015** | Efficacy | Low | Low | | Low | Low | Low | Low | ·· | Low |
|  | Tolerability | ·· | ·· | | ·· | ·· | ·· | ·· | ·· | ·· |
|  | Safety | ·· | ·· | | ·· | ·· | ·· | ·· | ·· | ·· |
| **Poespoprodjo 2022** | Efficacy | Low | Moderate | | Low | Low | Low | Low | ·· | Moderate |
|  | Tolerability | Low | Moderate | | Low | Low | Moderate | Low | ·· | Moderate |
|  | Safety | ·· | ·· | | ·· | ·· | ·· | ·· | ·· | ·· |

·· (not applicable in this IPD or not eligible in the specified analysis)


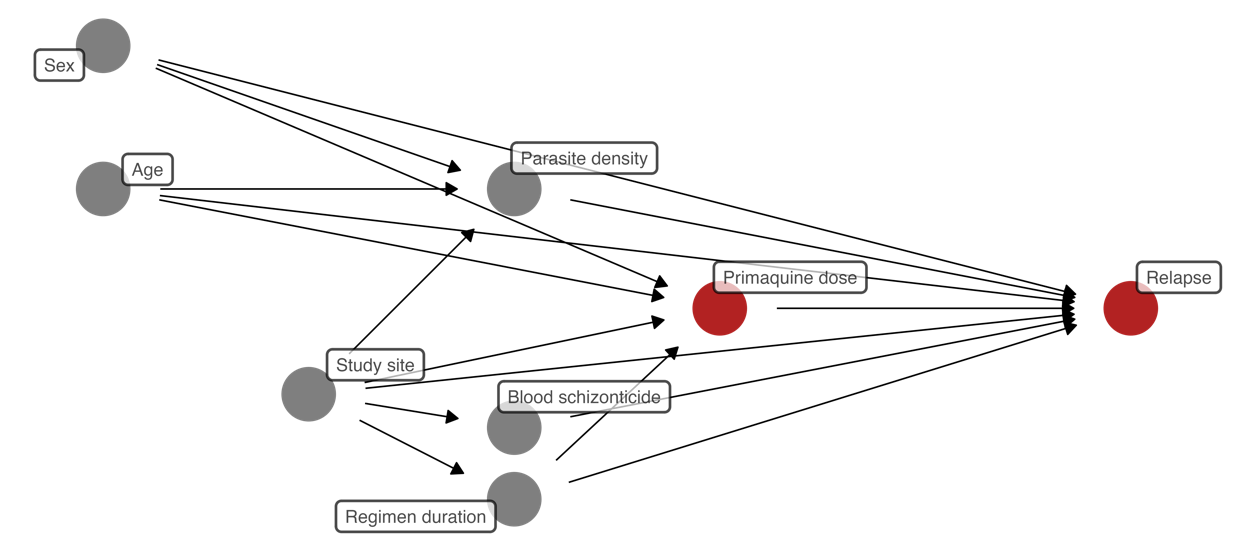


**Figure S1. Causal directed acyclic graph showing the relationship between the two factors of interest (red nodes): primaquine mg/kg total dose and the risk of P. vivax relapse**

Under this causal framework, age, sex, and study site are considered potential confounders of the dose–relapse association. These variables may act as proxies for underlying host factors (e.g., body weight, acquired immunity, treatment-seeking behaviour, CYP2D6 polymorphisms) and geographical or parasite-related characteristics (e.g., strain relapse periodicity, transmission intensity, blood-stage drug resistance) that influence both primaquine dosing and relapse risk. Parasite density, blood schizontocidal treatment, and regimen duration are included as factors that may affect relapse risk and/or influence treatment allocation. The directed acyclic graph informed selection of adjustment variables in the regression models.


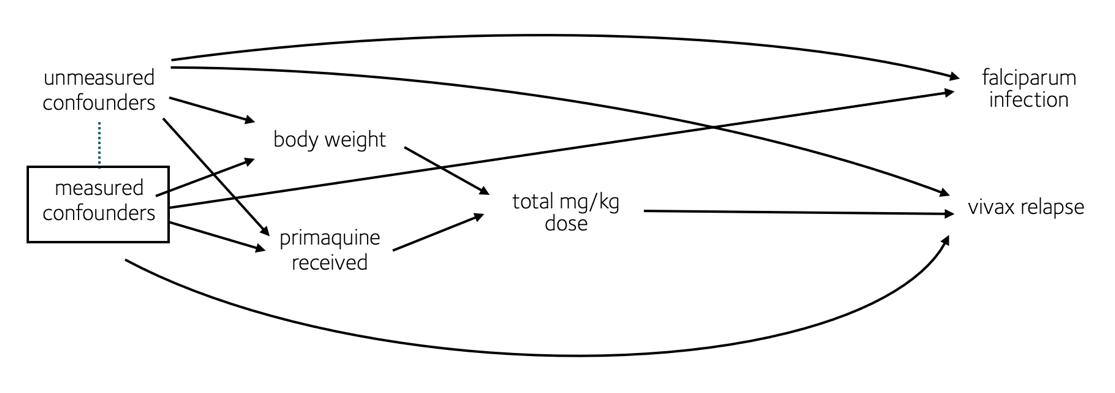


A


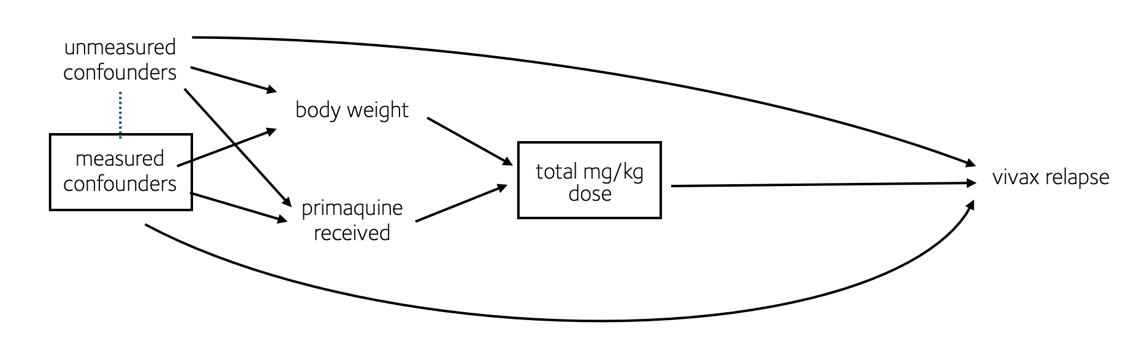


B

**Figure S2. Causal directed acyclic graphs illustrating the use of P. falciparum infection and body weight as negative controls to detect residual confounding**

A. Outcome negative control: The presence of P. falciparum re-infection in patients treated for P. vivax mono-infection during follow-up in areas with local transmission should be independent of the total primaquine dose received, provided that measured confounding is adequately adjusted for. However, if substantial residual confounding is not sufficiently controlled (either due to inadequate adjustment of measured confounding or the presence of unmeasured confounding), a spurious causal effect of primaquine dose on the risk of P. falciparum infection may become apparent.

B. Exposure negative control: Among patients with P. vivax who received no primaquine (thereby excluding the essential anti-hypnozoite component of treatment) the risk of P. vivax relapse should be independent of the total primaquine dose received, provided that measured confounding is adequately adjusted for. However, if substantial residual confounding is not sufficiently controlled (either due to inadequate adjustment of measured confounding or the presence of unmeasured confounding) a spurious causal effect of body weight on the risk of P. vivax relapse may become apparent.

A node within a square represents an adjustment, either through model stratification or restriction in our analysis. A dotted line indicates that two nodes may be related due to some common causes.


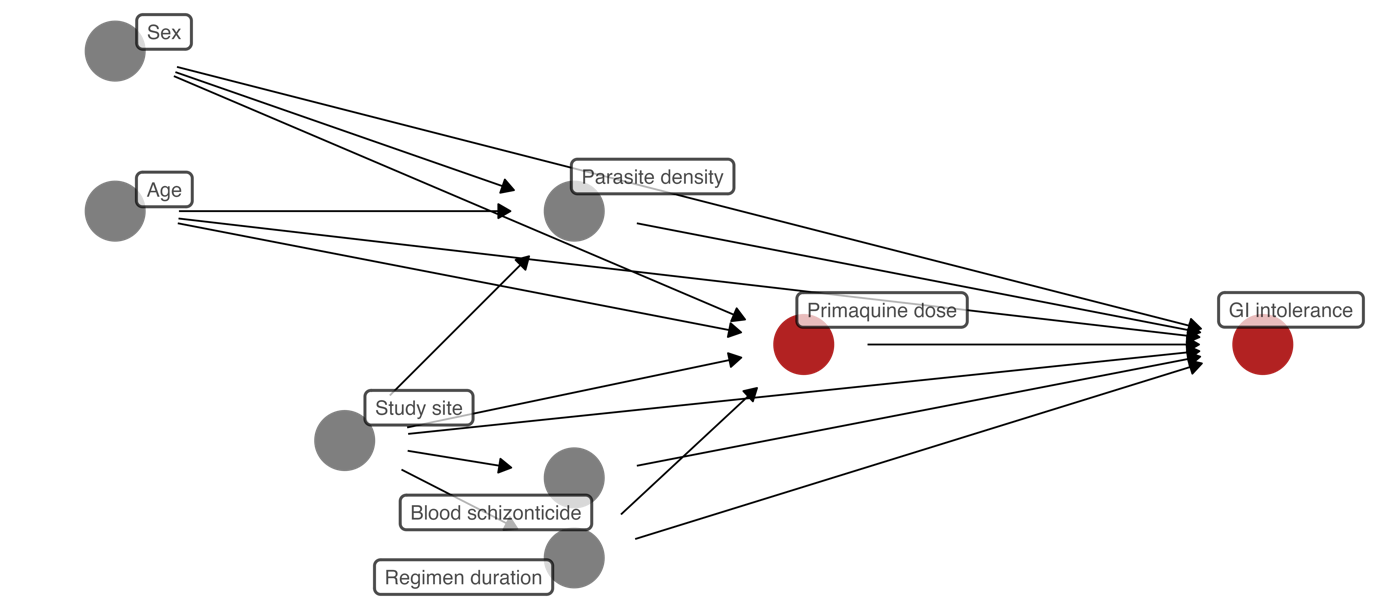


**Figure S3. Causal directed acyclic graph showing the relationship between the two factors of interest (red nodes): primaquine mg/kg daily dose and the risk of gastrointestinal (GI) discomfort**

Under this causal framework, age, sex, and study site are considered potential confounders of the dose–GI causal association. These variables may act as proxies for underlying host characteristics (e.g., body weight, treatment adherence, CYP2D6 polymorphisms) and contextual or geographical factors that influence both dosing and susceptibility to gastrointestinal symptoms. The directed acyclic graph informed covariate selection for adjustment in the tolerability analyses.


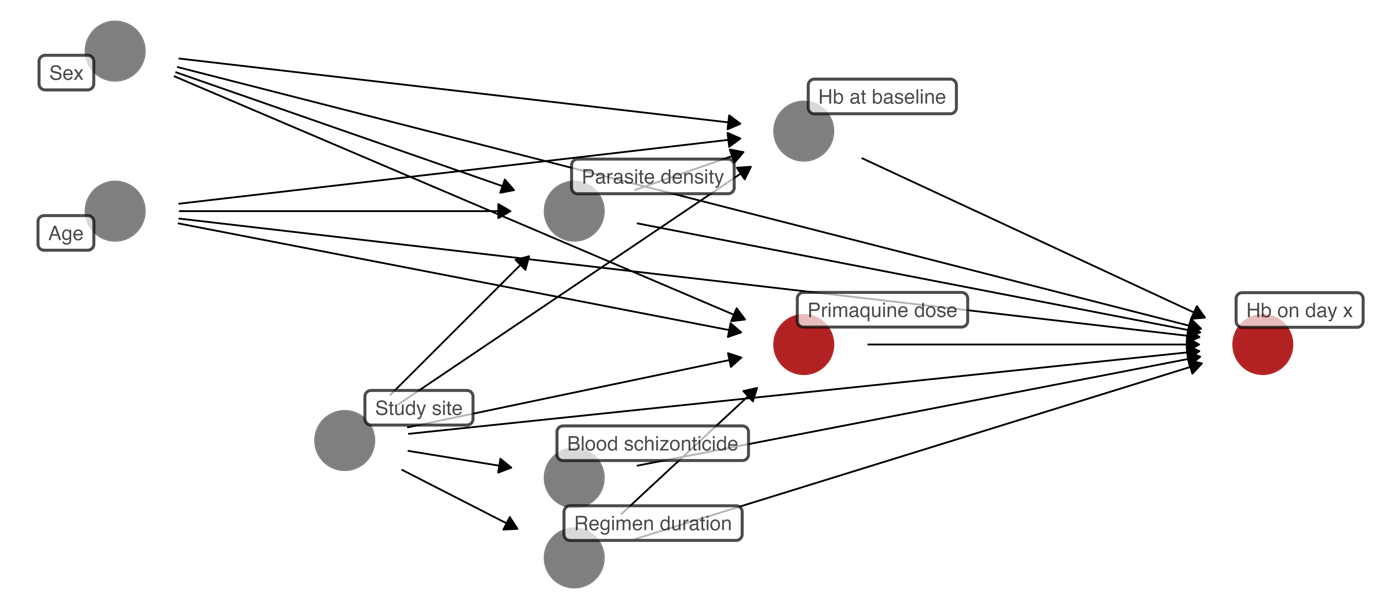


**Figure S4. Causal directed acyclic graph showing the relationship between the two factors of interest (red nodes): primaquine mg/kg daily dose and haemolysis**

Under this causal framework, age, sex, and study site are considered potential confounders of the dose–haemolysis association. These variables may act as proxies for underlying host factors (e.g., body weight, baseline haemoglobin, G6PD status, treatment behaviours, CYP2D6 polymorphisms) and geographical or epidemiological characteristics that influence both dosing and susceptibility to haemolysis. The directed acyclic graph informed covariate selection for adjustment in the haematological safety analyses.


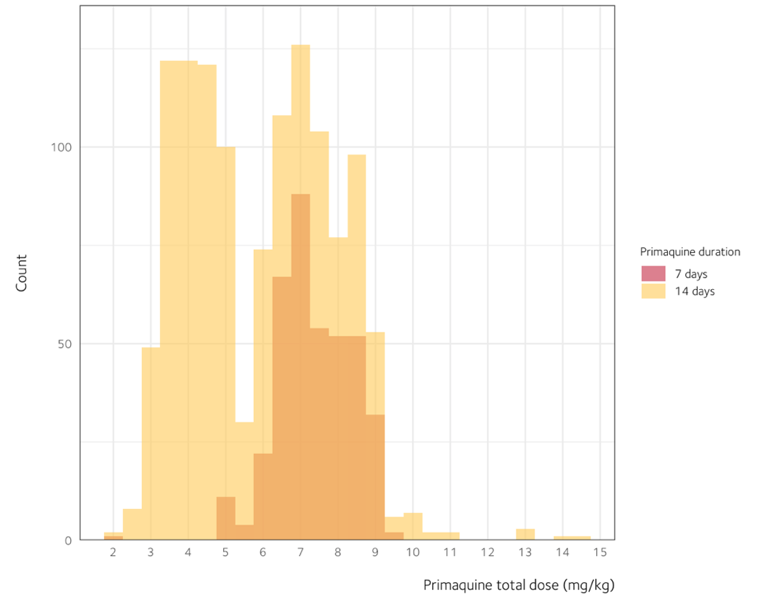


**Figure S5. Distribution of primaquine total dose by primaquine duration**

In the 14-day primaquine regimen, the observed two peaks reflect the targeted total primaquine dose of 3·5 and 7 mg base per kg body weight.


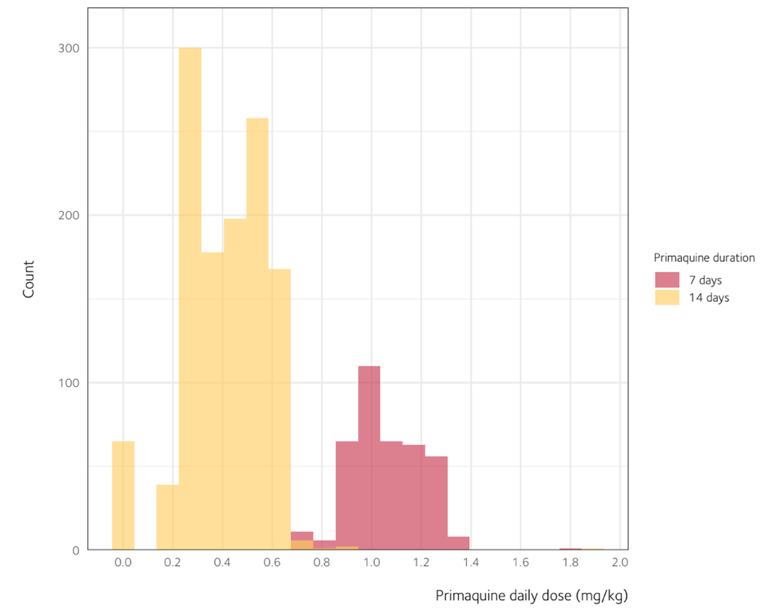


**Figure S6. Distribution of primaquine daily dose by primaquine regimen**

**Table S3. Patient characteristics by primaquine total dose group**

| **Characteristic** | **Primaquine total dose** | | | **Overall** |
| --- | --- | --- | --- | --- |
|  | **No primaquine**  **0 mg/kg** | **Low dose**  **2 to <5 mg/kg** | **High dose**  $\boldsymbol{\geq}$**5 mg/kg** |  |
| **Number of patients** | 196 | 464 | 1137 | 1797 |
| **Median age (years)** | 16 (9, 30) | 18 (11, 31) | 16 (9, 29) | 17 (10, 29) |
| **Age group** |  |  |  |  |
| Less than 5 years | 13 (6·6%) | 50 (11%) | 109 (9·6%) | 172 (9·6%) |
| 5 to 14 years | 76 (39%) | 136 (29%) | 393 (35%) | 605 (34%) |
| At least 15 years | 107 (55%) | 278 (60%) | 635 (56%) | 1020 (57%) |
| **Male sex** | 111 (57%) | 258 (56%) | 687 (60%) | 1056 (59%) |
| **Body weight (kg)** | 45 (23, 56) | 45 (26, 54) | 46 (23, 59) | 45 (24, 57) |
| **Baseline parasite density (per μL)** | 3831 (1200, 9543) | 1160 (320, 4130) | 3344 (830, 7574) | 2793 (630, 7130) |
| **Presence or recent history of fever** | 171 (87%) | 448 (97%) | 904 (89%) | 1523 (91%) |
| Number of missing data | 0 | 1 | 119 | 120 |
| **Schizontocidal drug** |  |  |  |  |
| Dihydroartemisinin-Piperaquine | 196 (100%) | 248 (53%) | 988 (87%) | 1432 (80%) |
| Artesunate-Amodiaquine | 0 (0%) | 196 (42%) | 42 (3·7%) | 238 (13%) |
| Artesunate-Pyronaridine | 0 (0%) | 1 (0·2%) | 59 (5·2%) | 60 (3·3%) |
| Chloroquine | 0 (0%) | 19 (4·1%) | 9 (0·8%) | 28 (1·6%) |
| Quinine | 0 (0%) | 0 (0%) | 39 (3·4%) | 39 (2·2%) |
| **Primaquine total dose (mg base/kg)** | 0·00 (0·00, 0·00) | 4·04 (3·50, 4·38) | 7·21 (6·52, 8·12) | 6·38 (4·12, 7·53) |
| **Primaquine daily dose (mg base/kg)** | 0·00 (0·00, 0·00) | 0·29 (0·25, 0·31) | 0·58 (0·48, 0·96) | 0·47 (0·28, 0·63) |
| **Primaquine duration** |  |  |  |  |
| No primaquine | 196 (100%) | 0 (0%) | 0 (0%) | 196 (11%) |
| 7 days | 0 (0%) | 5 (1·1%) | 380 (33%) | 385 (21%) |
| 14 days | 0 (0%) | 459 (99%) | 757 (67%) | 1216 (68%) |
| **Primaquine dose calculation** |  |  |  |  |
| No primaquine | 196 (100%) | 0 (0%) | 0 (0%) | 196 (11%) |
| Actual dosing | 0 (0%) | 42 (9·1%) | 1037 (91%) | 1079 (60%) |
| Protocol dosing | 0 (0%) | 422 (91%) | 100 (8·8%) | 522 (29%) |
| **Primaquine supervision** |  |  |  |  |
| No primaquine | 196 (100%) | 0 (0%) | 0 (0%) | 196 (11%) |
| Unsupervised | 0 (0%) | 129 (28%) | 77 (6.8%) | 206 (11%) |
| Partially supervised | 0 (0%) | 9 (1.9%) | 80 (7.0%) | 89 (5.0%) |
| Fully supervised | 0 (0%) | 326 (70%) | 980 (86%) | 1306 (73%) |
| **Baseline haemoglobin level (g/dL)** | 12·75 (11·60, 14·10) | 11·50 (10·30, 12·70) | 12·60 (11·30, 14·00) | 12·30 (11·00, 13·70) |
| Number of missing data | 0 | 2 | 0 | 2 |
| **Day 7 methaemoglobin level (%)** | NA | 3·9 (2·7, 5·9) | 6·9 (4·2, 10·3) | 6·0 (3·5, 9·4) |
| Number of missing data | 196 | 213 | 502 | 911 |
| **Transmission intensity** |  |  |  |  |
| Low | 0 (0%) | 0 (0%) | 0 (0%) | 0 (0%) |
| Moderate | 196 (100%) | 306 (66%) | 948 (83%) | 1450 (81%) |
| High | 0 (0%) | 158 (34%) | 189 (17%) | 347 (19%) |
| **Origin of infections** |  |  |  |  |
| Within Papua | 0 (0%) | 139 (30%) | 313 (28%) | 452 (25%) |
| Outside Papua | 196 (100%) | 325 (70%) | 824 (72%) | 1345 (75%) |
| **Study duration** |  |  |  |  |
| At least 180 days | 196 (100%) | 307 (66%) | 1104 (97%) | 1607 (89%) |
| Less than 180 days | 0 (0%) | 157 (34%) | 33 (2·9%) | 190 (11%) |

Numbers are in median (first quartile [Q1], third quartile [Q3]) or frequency (percentage). NA, not available; kg, kilogram; μL, microlitre; g, gram; dL, decilitre; mg, milligram. Transmission intensity was categorised as low (<1 case per 1000 person-years), moderate (1 case to <10 cases per 1000 person-years), and high (≥10 cases per 1000 person-years) according to subnational malaria incidence estimates for the median year of study enrolment.^49^ Patients contributing to the tolerability and safety datasets, as well as any sensitivity or restricted analyses, were subsets of the dataset shown in this table.

**Table S4. Studies included in analysis**

| **Paper** | **Study site** | **Latitude** | **Longitude** | **Year start** | **Year end** | **MAP incidence rate (per 1000 person-years)** | **Transmission intensity#** | **Relapse periodicity§** |
| --- | --- | --- | --- | --- | --- | --- | --- | --- |
| **Hasugian 2007^24^** | Timika | –4·61 | 136·85 | 2005 | 2005 | 22.61 | High | High |
| **Pasaribu 2013^14^** | Tanjung Leidong | 2·77 | 99·98 | 2011 | 2011 | 2·75 | Moderate | High |
| **Sutanto 2013^13^** | Lumajang | –8·13 | 113·22 | 2010 | 2011 | 36·88* | High | High |
| **Lidia 2015^25^** | Kupang | –10·18 | 123·61 | 2013 | 2013 | 15·22 | High | High |
| **Nelwan 2015^11^** | Sragen | –7·42 | 111·02 | 2013 | 2013 | 42·44* | High | High |
| **Taylor 2019^15^** | Hanura | –5·53 | 105·24 | 2015 | 2017 | 1·01 | Moderate | High |
| **Taylor 2019^15^** | Tanjung Leidong | 2·77 | 99·98 | 2015 | 2017 | 1·03 | Moderate | High |
| **Poespoprodjo 2022^12^** | Timika | –4·45 | 136·98 | 2015 | 2018 | 56·43 | High | High |

MAP malaria atlas project. § Relapse periodicity was categorised as high (median relapse periodicity of 47 days or less) and low (median relapse periodicity of more than 47);^52^ # Transmission intensity was categorised as low (<1 case per 1000 person-years), moderate (1 case to <10 cases per 1000 person-years), and high (≥10 cases per 1000 person-years) according to subnational malaria incidence estimates for the median year of study enrolment.^49^ * Based on the location where patients were infected by *P. vivax* in Papua.

**Table S5. Studies that were eligible for analysis but not included for data pooling**

| **Characteristic** | **Study** | | |
| --- | --- | --- | --- |
|  | **Maguire et al.^27^*** | **Arcelia et al.^28^** | **Sutanto et al.^26^** |
| **Year published** | 2006 | 2023 | 2023 |
| **Number of treatment arms** | 2 | 1 | 3 |
| **Region origin of infection** | Papua | Outside Papua | Papua |
| **Number of sites** | 1 | 1 | 2 |
| **Follow-up (days)** | 28 | 28 | 180 |
| **Randomised** | Yes | No | Yes |
| **Recruitment period** | 1996–1999 | 2019–2020 | 2018–2019 |
| **Treatment arms** | (1) Chloroquine + 14-day, low dose primaquine  (2) Mefloquine + 14-day, low dose primaquine | (1) Dihydroartemisinin-Piperaquine + 14-day, low dose primaquine | (1) Dihydroartemisinin-Piperaquine  (2) Dihydroartemisinin-Piperaquine + 14-day, low dose primaquine  (3) Dihydroartemisinin-Piperaquine + Tafenoquine |
| ***P. vivax* patients enrolled** | 125* | 60 | 150 |
| **Treated with primaquine** | 125* | 60 | 50 |
| **Primaquine supervision** | NA | NA | Yes |
| **Sex (primaquine receiving arm)** | 68% Male* | NA | 100% Male |
| **Age (in years, primaquine receiving arm)** | Mean = 22·3* | 30 Children (2 to 18 years)  30 Adults | Mean = 28·6 |
| **Reason for exclusion** | Missing minimum data | Data not available  by 28 February 2025 | Data not available  by 28 February 2025 |

* Based on contributed IPD that may include both randomised trials and prospective cohorts not part of the trial itself. NA, not available; IPD, individual patient data.

**Table S6. Comparison of patient characteristics who received primaquine between included studies and eligible but not available studies**

| **Characteristic** | **Included studies (n = 7)** | **Eligible but not included studies (n = 3)** |
| --- | --- | --- |
| **Region origin of infection, studies (percentage)** | | |
| Papua | 2 (28·6%) | 2 (66·7%) |
| Outside Papua | 5 (71·4%) | 1 (33·3%) |
| **Year of enrolment, studies (percentage)** | | |
| Pre-2015 | 5 (71·4%) | 1 (33·3%) |
| 2015-2019 | 2 (28·6%) | 2 (66·7%) |
| **Follow up duration in days, studies (percentage)** | | |
| 42 | 2 (28·6%) | 2 (66·7%) |
| >42 to <120 | 0 (0%) | 0 (0%) |
| 120 | 0 (0%) | 0 (0%) |
| >120 | 5 (71·4%) | 1 (33·3%) |
| **Age (weighted-average years)^#,$^** | 16·9^*^ | 25·7^§^ |
| **Male (weighted percentage)^#^** | 59·6%^*^ | 85%^§^ |

# Weights approximated by the numbers of *P. vivax* patients treated with primaquine. * Based on included participants in this pooled analysis (n = 1797). $ Study-specific average is the mean or median in vivax patients treated with primaquine. § Based on two studies from Table S5 for which the required summary statistics were available.


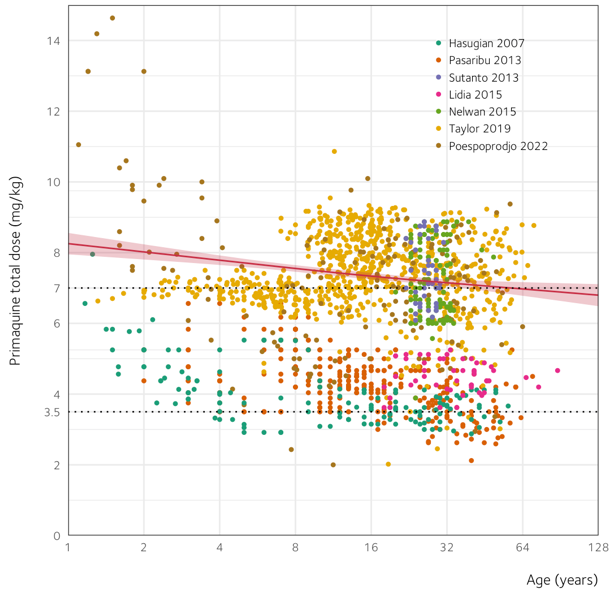

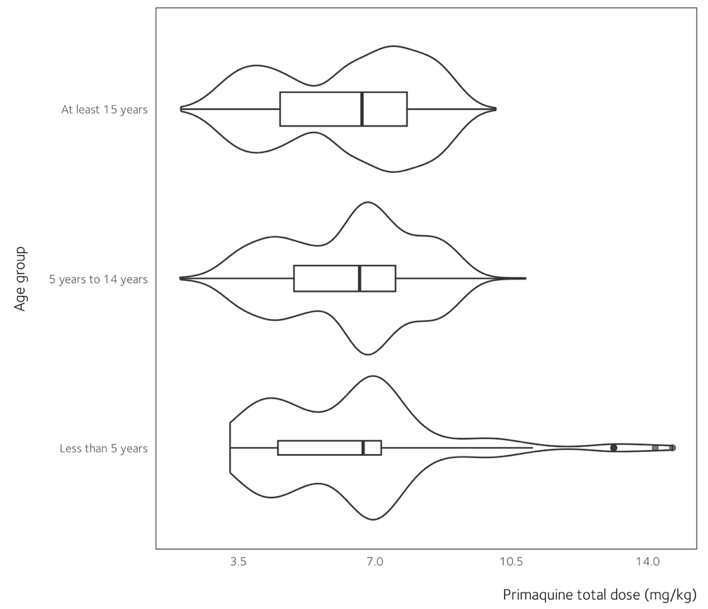


B

A

**Figure S7. Primaquine total dose received across different patient age**

A. While the median average across the three age groups was similar, patients under five years old were more likely to receive a higher total dose (relative to a target dose) than older patients, as also depicted in panel B. In B, each dot represents an individual patient. The red curve denotes a fitted regression line between age and primaquine total dose. Dotted line represents the target dose of 3·5 or 7 mg base per kg body weight. This may reflect the relatively greater difficulty of administering primaquine in very young patients. For panel B, the horizontal axis is shown on a logarithmic scale.


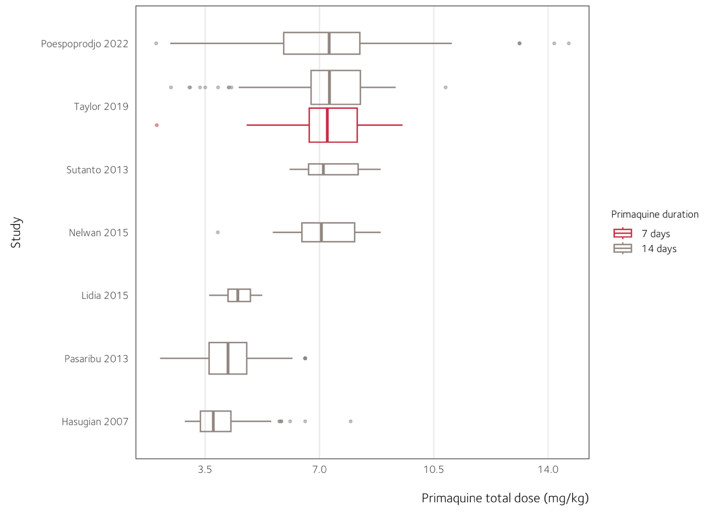

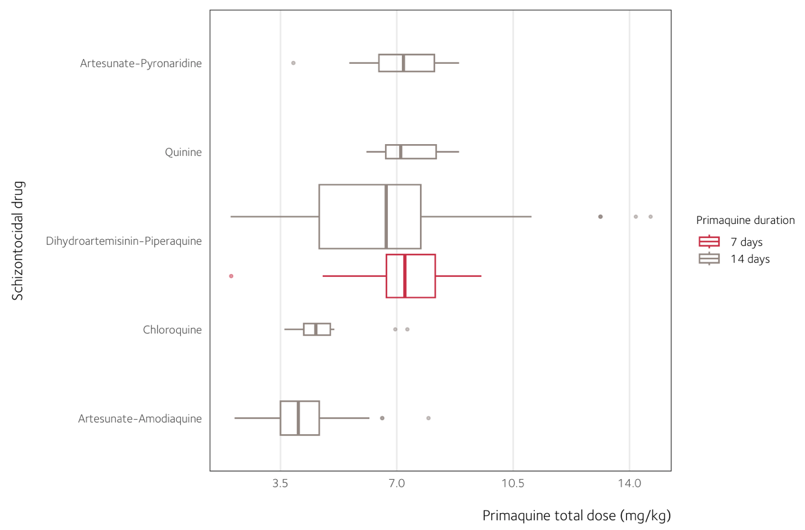


B

A

**Figure S8. Primaquine total dose by schizontocidal drug (A) and study (B)**


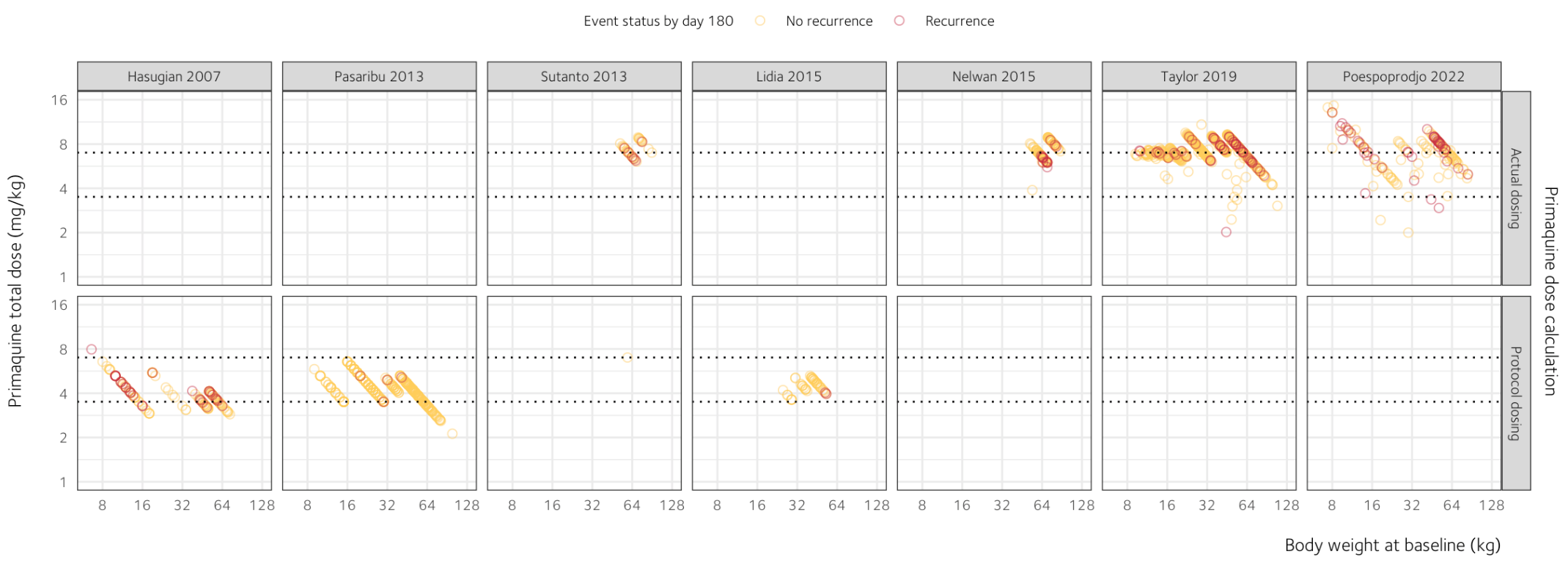


**Figure S9. Primaquine total dose by body weight in patients receiving primaquine**

Dotted line represents the total dose targets of 3·5 or 7 mg/kg. For Hasugian 2007 and Lidia 2015, the follow up duration was less than 180 days. Event status indicates whether the patient developed a *P. vivax* recurrence. Horizontal and vertical axes are shown on a logarithmic scale.


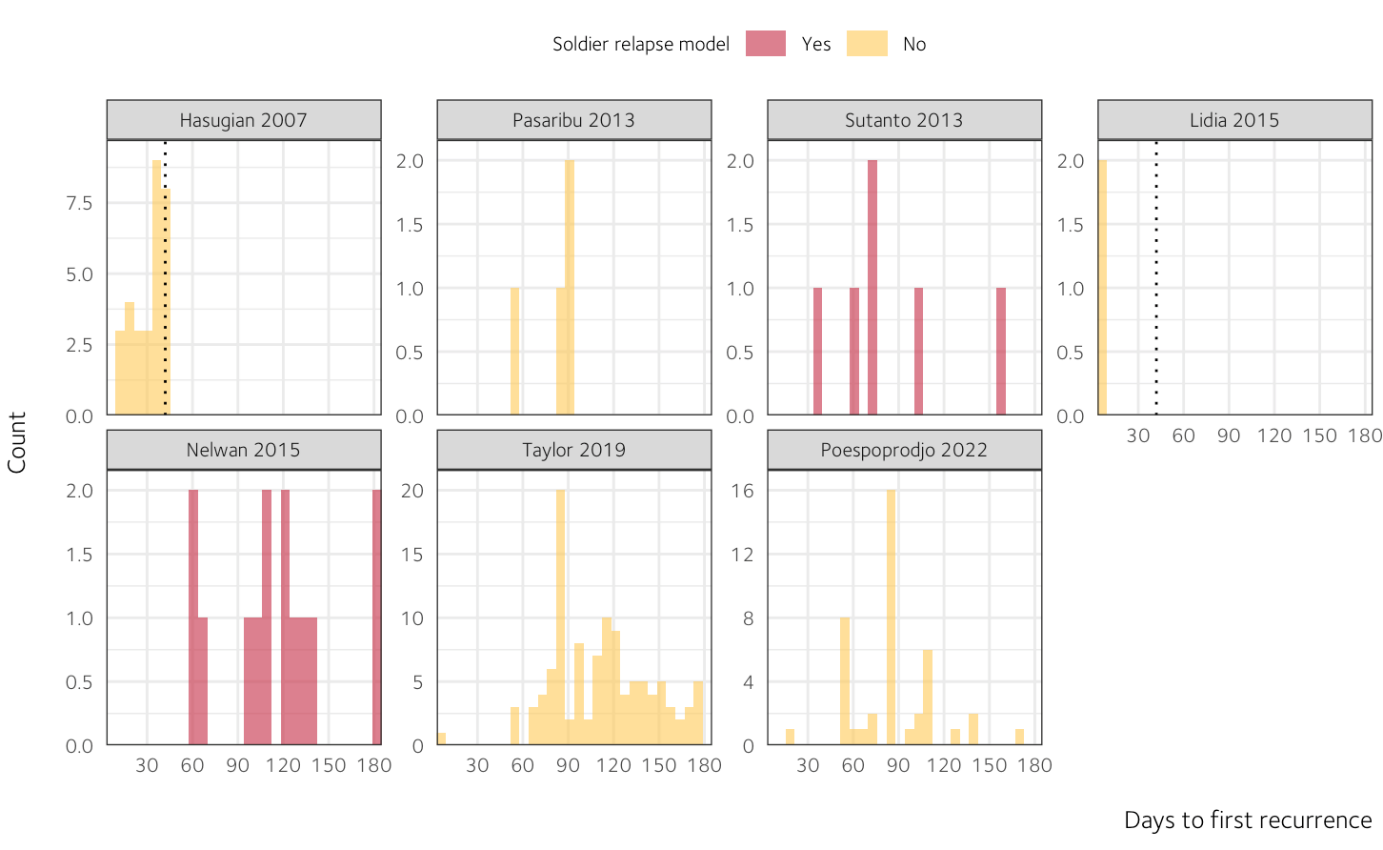


**Figure S10. Distributions of the days to first P. vivax recurrence by study**

For Hasugian 2007 and Lidia 2015, the dotted line represents the end of active follow up. The other studies monitored patients for at least 180 days.

**Table S7. Model estimates underlying the main results**

| **Model** | **Coefficient** | **Standard error** |
| --- | --- | --- |
| **Efficacy (Cox proportional hazards regression)** | | |
| Primaquine total dose (spline term 1) | –0·3185 | 0·0416 |
| Primaquine total dose (spline term 2) | 0·1794 | 0·0494 |
| Log baseline parasite density (linear term) | 0·2154 | 0·0514 |
| Age (linear term) | –0·0305 | 0·0108 |
| Age × time | 0·0003 | 0·0001 |
| **Tolerability (Poisson regression)** | | |
| Primaquine daily dose (spline term 1) | 1·9431 | 0·7109 |
| Primaquine daily dose (spline term 2) | –1·2393 | 0·9385 |
| Log baseline parasite density (linear term) | 0·0380 | 0·0654 |
| Age (linear term) | 0·0214 | 0·0064 |
| Sex (male vs. female) | –0·2056 | 0·2055 |
| Intercept | –4·0943 | 0·6799 |
| **Safety (linear regression)** | | |
| Primaquine daily dose (linear term) | –8·1201 | 6·4840 |
| Log_2_ G6PD activity (spline term 1) | 0·5708 | 0·9312 |
| Log_2_ G6PD activity (spline term 2) | 0·8153 | 2·7176 |
| Log_2_ G6PD activity (spline term 3) | –8·5553 | 10·5350 |
| Baseline haemoglobin (linear term) | –0·3670 | 0·0207 |
| Age (linear term) | 0·0006 | 0·0024 |
| Sex (male vs. female) | 0·2286 | 6·9952 |
| Study site (Tanjung Leidong vs. Hanura) | 0·0877 | 0·0735 |
| Baseline parasite density (linear term) | 0·0086 | 0·0208 |
| Primaquine daily dose × log_2_ G6PD activity (spline 1) | 1·3102 | 1·0217 |
| Primaquine daily dose × log_2_ G6PD activity (spline 2) | –5·9293 | 3·2047 |
| Primaquine daily dose × log_2_ G6PD activity (spline 3) | 27·0140 | 12·6741 |
| Primaquine daily dose × sex | 11·8332 | 8·4664 |
| log_2_ G6PD activity (spline 1) × sex | 0·0355 | 1·1027 |
| log_2_ G6PD activity (spline 2) × sex | –1·3898 | 3·4900 |
| log_2_ G6PD activity (spline 3) × sex | 10·0486 | 14·1048 |
| Primaquine daily dose × log_2_ G6PD activity (spline 1) × sex | –1·8645 | 1·3382 |
| Primaquine daily dose × log_2_ G6PD activity (spline 2) × sex | 4·9666 | 4·4386 |
| Primaquine daily dose × log_2_ G6PD activity (spline 3) × sex | –20·2518 | 18·0407 |
| Intercept | –0·6788 | 5·9135 |

In the efficacy model, to relax certain modelling assumptions (e.g., proportional hazards and baseline hazards across sites), study site and sex were specified as stratification variables, and an age–time interaction term was included. Coefficient estimates from the Cox and Poisson models represent log hazard ratios and log risk ratios, respectively.


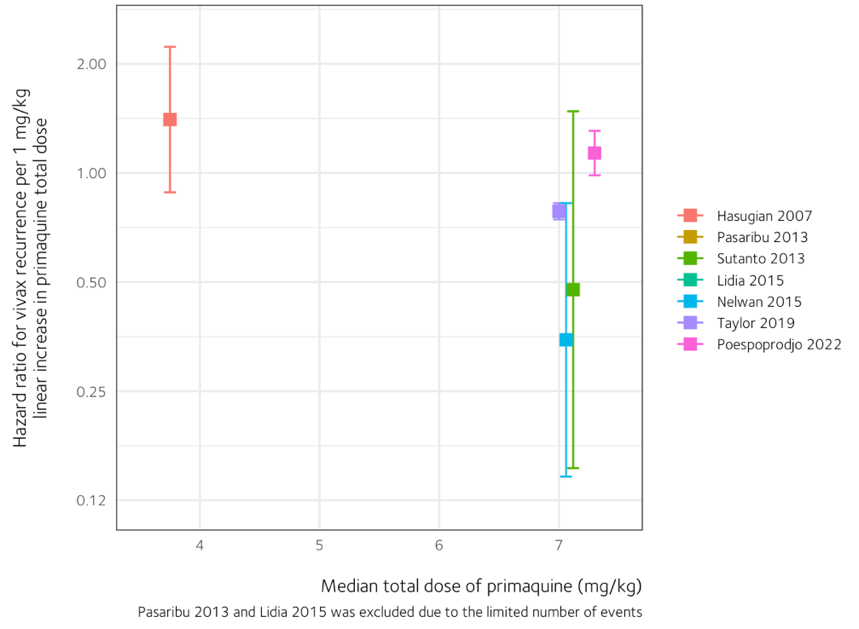

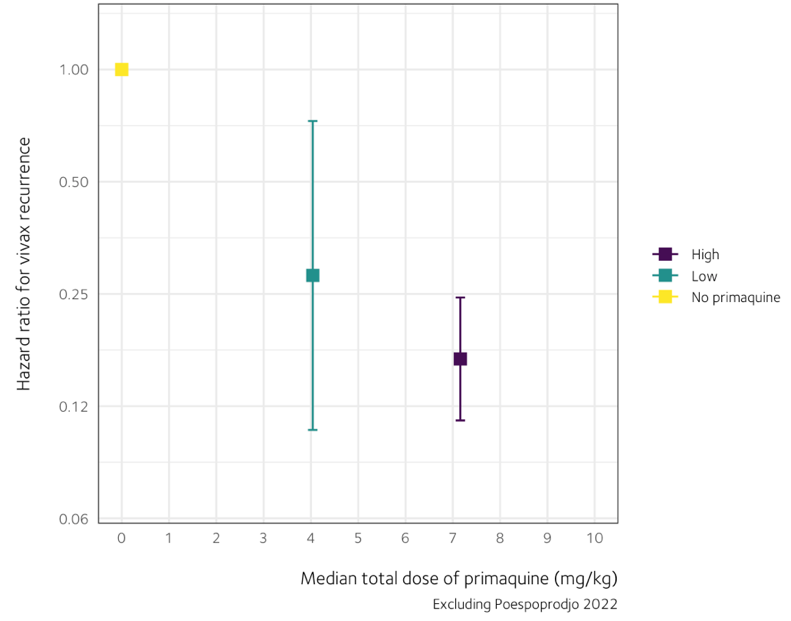

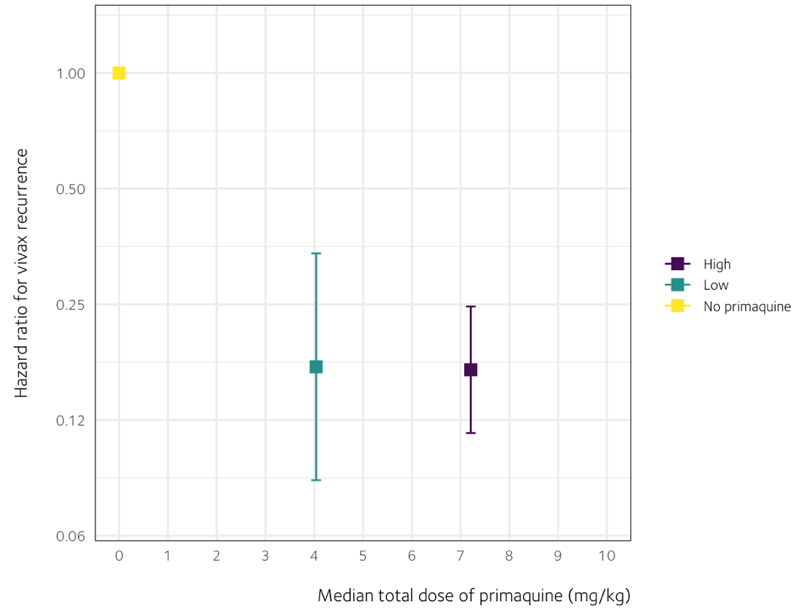


A

B

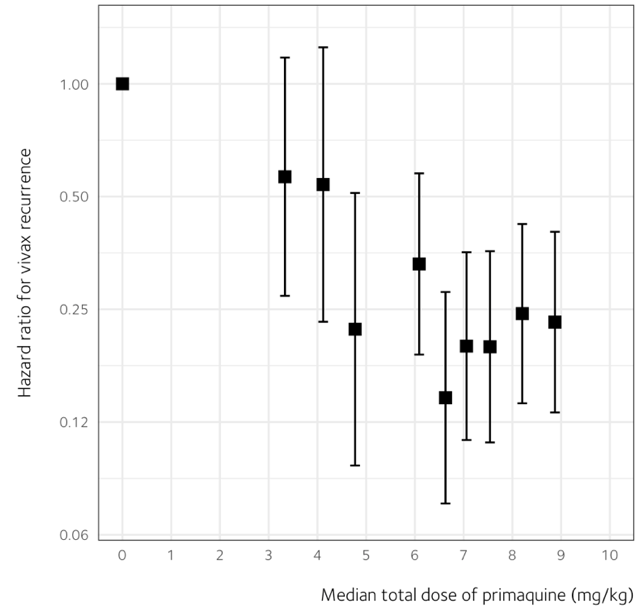


D

C


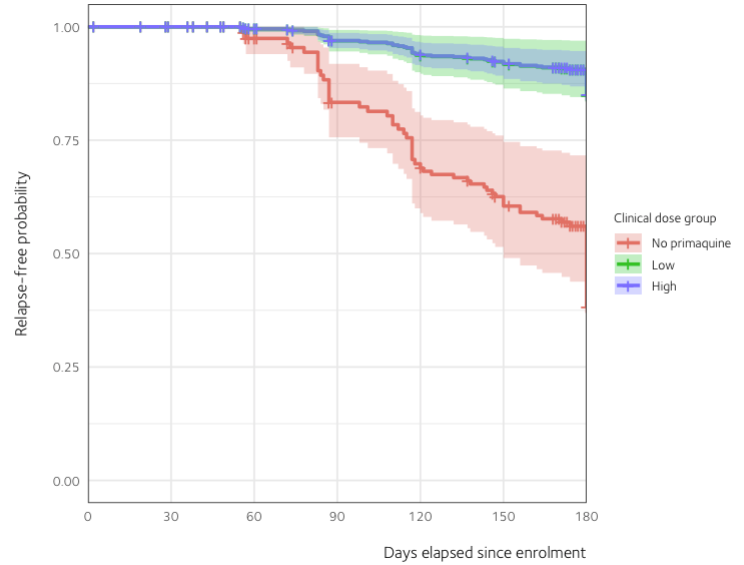

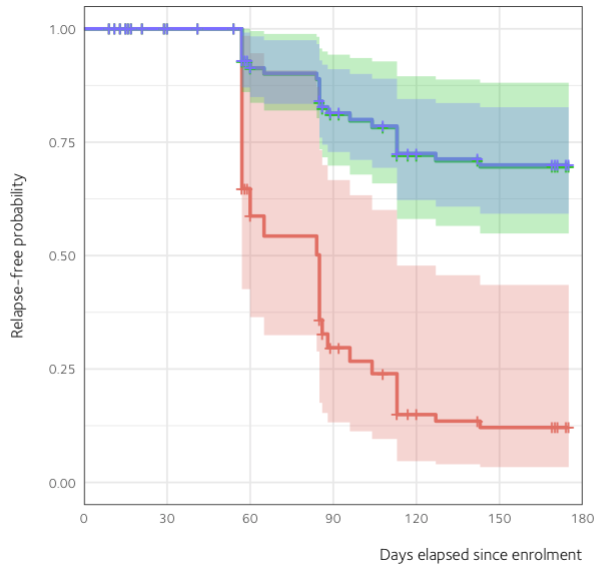


F

E

**Figure S11. P. vivax recurrence at day 180 by different groups**

By (A) clinical total dose group in the primary efficacy dataset, n = 1797; (B) Clinical total dose group excluding the cluster randomised trial,^12^ n = 1641; (C) Decile with 10 equal sized groups in the primary efficacy dataset, n = 1797; and (D) Excluding two studies^14,25^ due to the limited number of events, n = 1415. The reference value, at which the hazard ratio equals one, was set at 0 mg/kg. The whisker shows 95% confidence intervals. Estimates were derived from a multivariable Cox proportional hazards model. The vertical axis for A–D is shown on a logarithmic scale. Based on the model underlying panel (A), panels (E) and (F) display the model-implied relapse-free probabilities in different total dose groups, separately as examples in Papua and Non-Papua settings, respectively. To estimate these survival probabilities, continuous covariates were set at their median values, and sex was set to male. This dose–response relationship was broadly consistent across alternative dose groupings and sensitivity analyses.


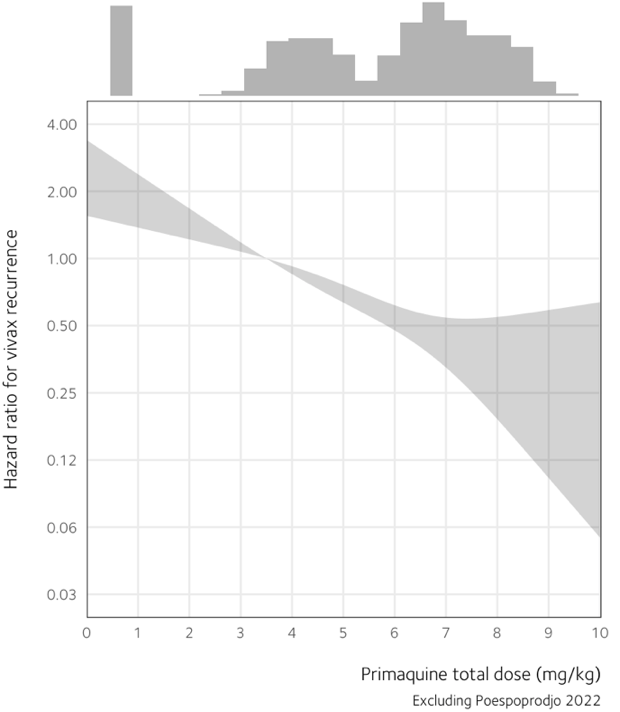


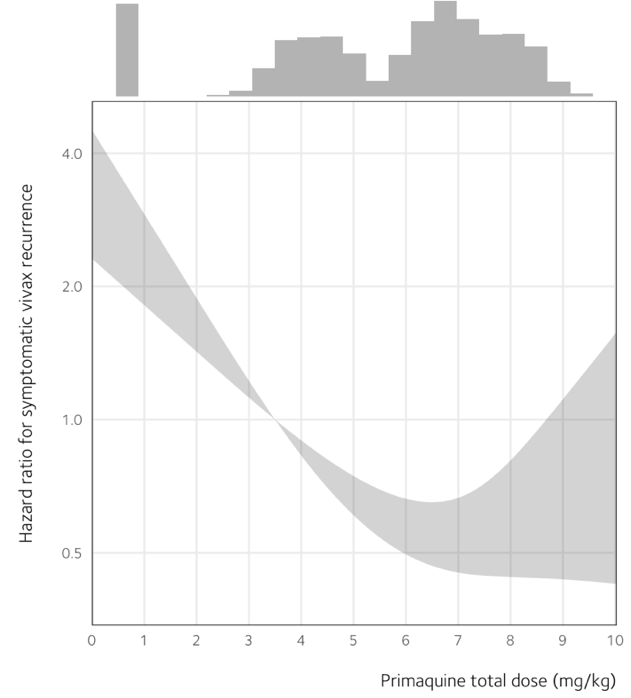


B

A


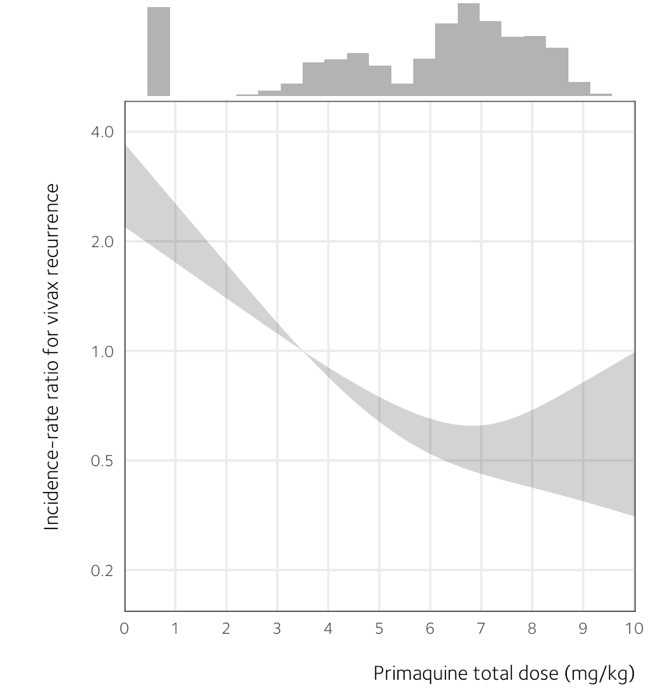


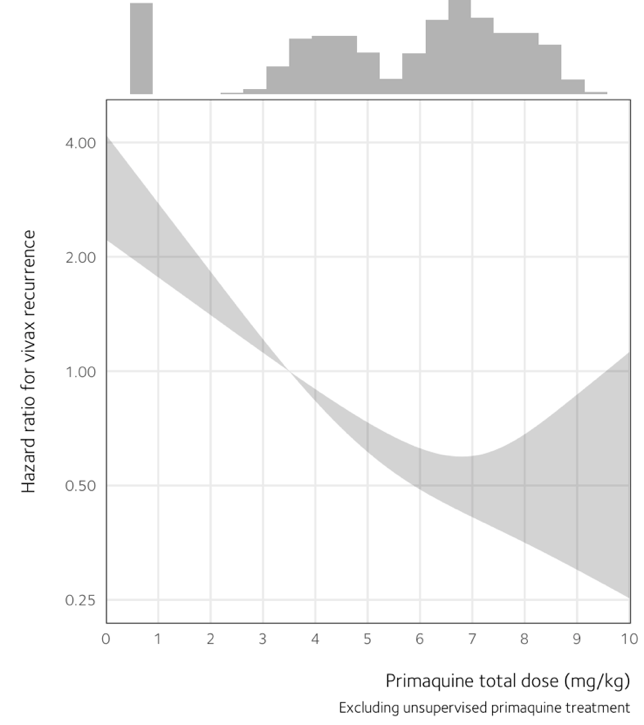


D

C

**Figure S12. Sensitivity analyses of the hazard ratio across primaquine total dose**

(A) Using symptomatic *P. vivax* recurrences as the endpoint event, n = 1794; (B) Excluding the cluster randomised trial,^12^ n = 1641; (C) Excluding unsupervised primaquine treatment, n = 1591; (D) Estimating the incidence rate ratio in patients followed for multiple *P. vivax* episodes, n = 1607. The reference value, at which the hazard ratio equals one, was set at the target low dose of 3·5 mg/kg. The shaded region shows 95% confidence intervals. Estimates were derived from a multivariable Cox proportional hazards model (A, B, C) or a multivariable Poisson model (D). A restricted cubic spline with three knots was specified on primaquine total dose to allow for a non-linear trend. The histogram along the top margin shows the distribution of primaquine daily doses in the model data, with the leftmost bar representing patients who were treated without primaquine (i.e., 0 mg/kg). The vertical axis is shown on a logarithmic scale. This dose–response relationship was broadly consistent across alternative dose groupings and sensitivity analyses.

**List S3. Negative control results**

Negative control methods for exposure (AHR of *P. vivax* recurrence associated with a 1 kg increase in body weight in patients treated without primaquine = 1.1; 95% CI 0.98 to 1.03; p = 0.60) and outcome (AHR of *P. falciparum* infection associated with a 1 mg/kg increase in primaquine total dose = 1.03; 95% CI 0.94 to 1.14; p = 0.48) suggest that residual confounding in our anti-relapse effect estimates was minimal.


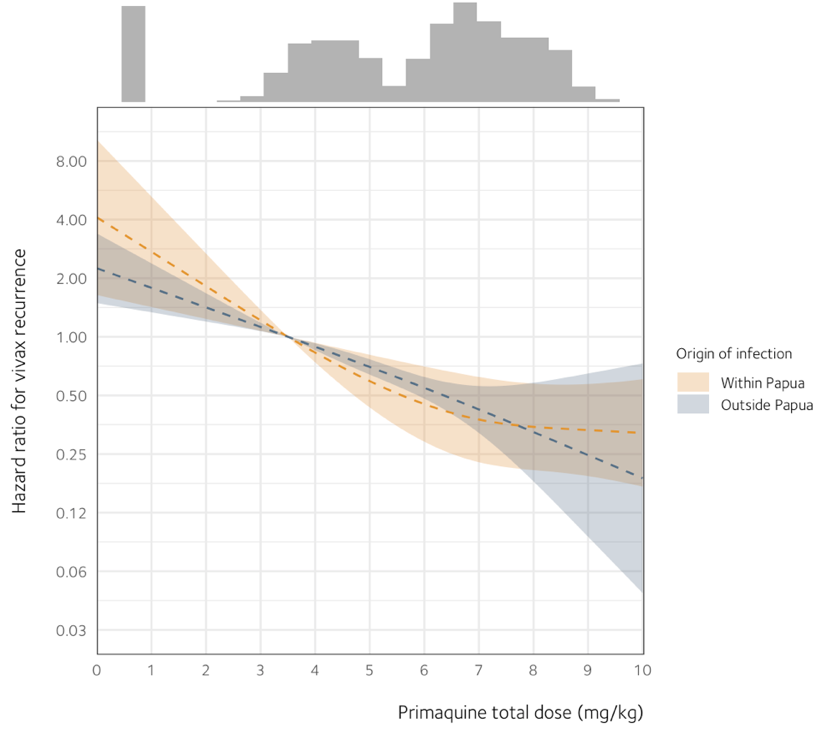


A


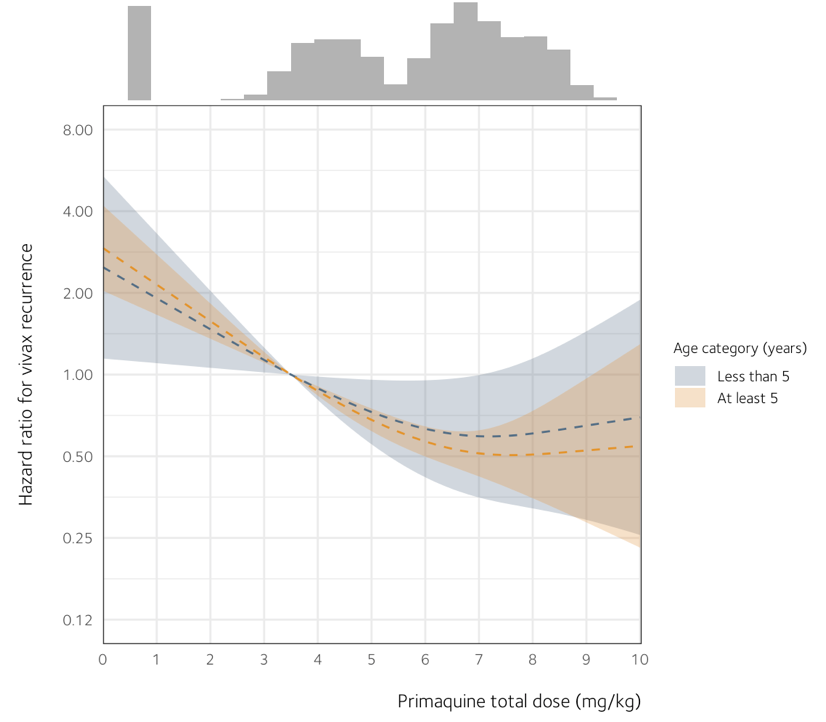


B

**Figure S13. Estimated hazard ratios of P. vivax recurrence at day 180, assuming effect modification by (A) origin of infection and (B) age category**

The reference value, at which the hazard ratio equals one, was set at the target low dose of 3·5 mg/kg. The shaded region shows 95% confidence intervals. Estimates were derived from a multivariable Cox proportional hazards model. A restricted cubic spline with three knots was specified on primaquine total dose to allow for a non-linear trend. The histogram along the top margin shows the distribution of primaquine daily doses in the model data (n = 1797), with the leftmost bar representing patients who were treated without primaquine (i.e., 0 mg/kg). The vertical axis is shown on a logarithmic scale.

**
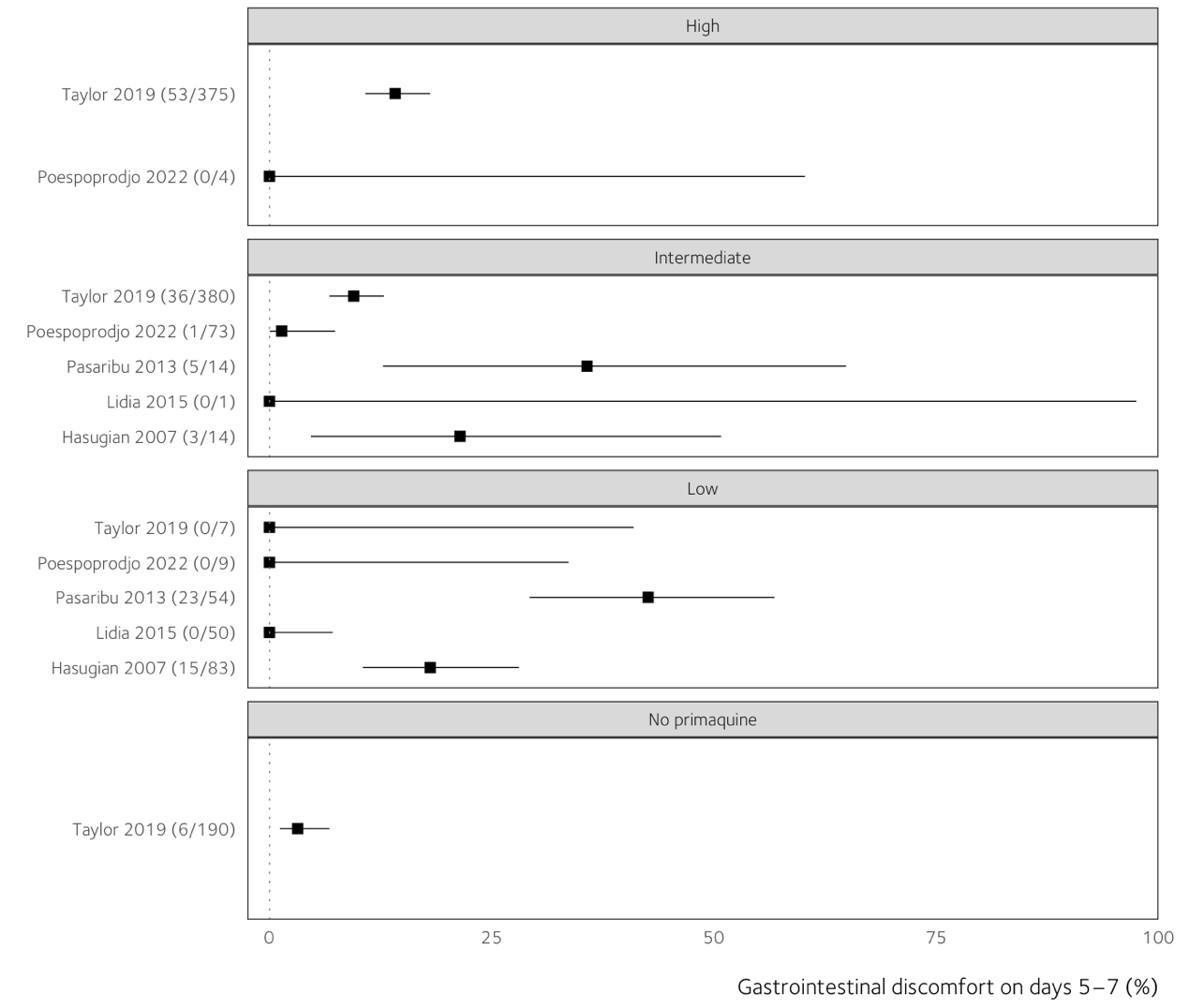
**

**Figure S14. Heterogeneity in percentages of gastrointestinal discomfort on days 5–7 by study and primaquine daily dose**

This variability may stem from the relatively subjective nature of assessing this endpoint. The solid square and horizontal line represent the point and 95% confidence interval estimates.

**Table S8. Pooled counts and percentages of patients who experienced gastrointestinal discomfort**

| **Primaquine daily dose** | **Days of observation** | | |
| --- | --- | --- | --- |
|  | **Baseline** | **1–2** | **5–7** |
| No primaquine | 0/0 (···) | 0/0 (···) | 6/190 (3·2%) |
| Low | 9/335 (2·7%) | 46/335 (13·7%) | 38/203 (18·7%) |
| Intermediate | 309/584 (52·9%) | 192/583 (32·9%) | 45/482 (9·3%) |
| High | 298/376 (79·3%) | 189/376 (50·3%) | 53/379 (14·0%) |
| Total | 616/1295 (47·6%) | 427/1294 (33·0%) | 142/1254 (11·3%) |


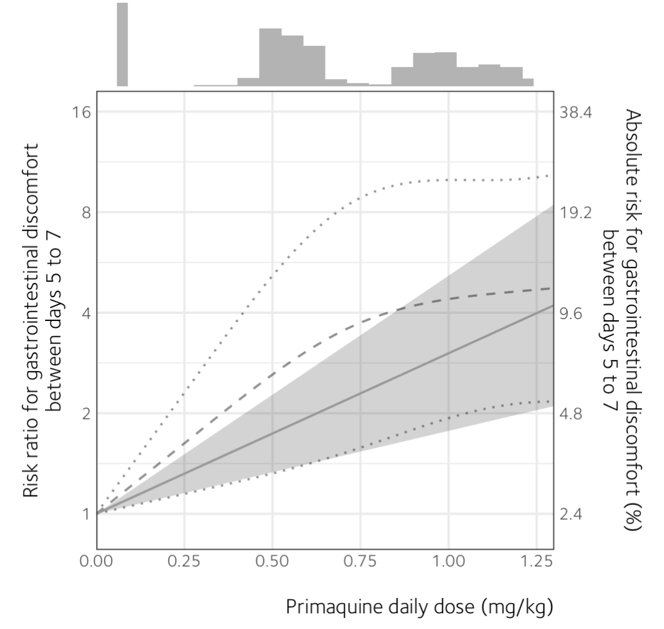


A


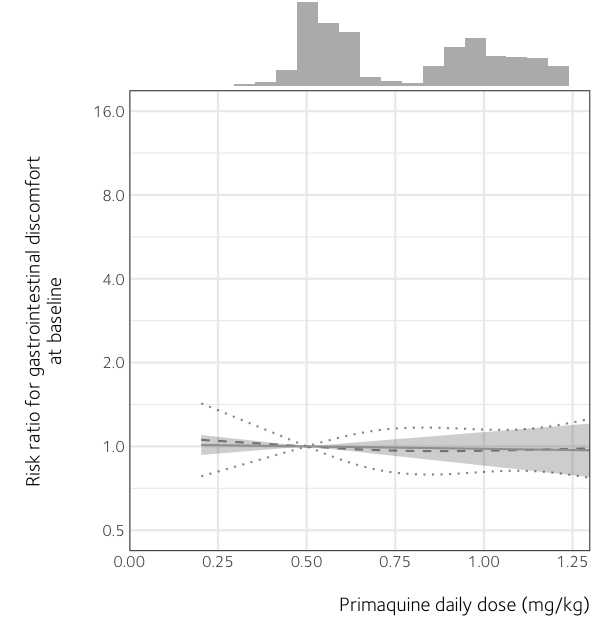

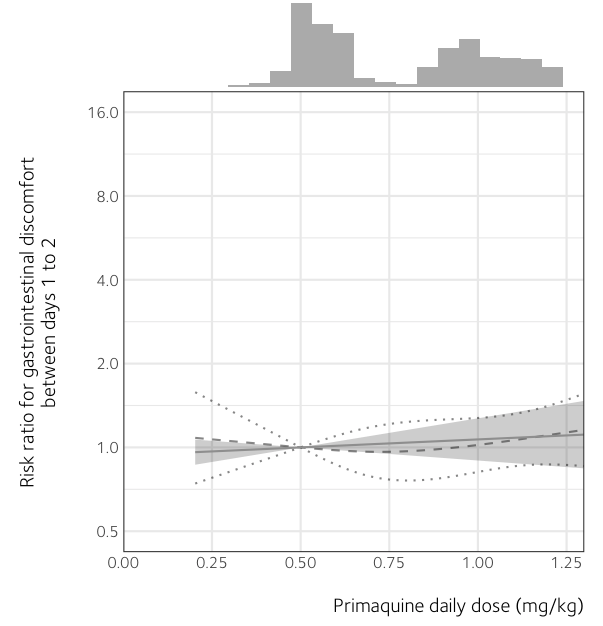


B

C

**Figure S15. Risk ratio of gastrointestinal discomfort (A) between days 5 to 7, (B) between days 1 to 2, and (C) at day 0 [baseline]**

(A) The reference value (RR = 1) was set at the primaquine daily dose of 0 mg/kg. Estimates were derived from a multivariable Poisson model (n = 952). Two different specifications for primaquine daily dose were shown to evaluate potential non-linearity across doses: linear trend (solid grey line with shaded region for 95% CI) and restricted cubic spline with three knots (dashed curve with two dotted curves representing the 95% CI limits). Inclusion of study site (p = 0.7) in a sensitivity analysis did not alter the effect-estimate for dose. The secondary y-axis displays the estimated absolute risk of gastrointestinal discomfort as a percentage. These values are derived by scaling the model-predicted risk ratios by the baseline risk. The histogram along the top margin shows the distribution of primaquine daily doses in the model data, with the leftmost bar representing patients who were treated without primaquine (i.e., 0 mg/kg). The vertical axis is shown on a logarithmic scale.

(B, C) The reference value, where the risk ratio equals one, was set at the primaquine daily dose of 0·5 mg/kg. Estimates were derived from a multivariable Poisson model (n in panel A = 767, n in panel B = 767). Two different specifications for primaquine daily dose were shown to evaluate potential non-linearity across doses: trend (solid grey line with shaded region for 95% CI) and restricted cubic spline with three knots (dashed curve with two dotted curves representing the 95% CI limits). The histogram along the top margin shows the distribution of primaquine daily doses in the model data, with the leftmost bar representing patients who were treated without primaquine (i.e., 0 mg/kg). The vertical axis is shown on a logarithmic scale. The y-axis is displayed up to a risk ratio of 16 to enable comparison with the primary gastrointestinal tolerability endpoint.

**Table S9. Pooled counts and percentages of patients who experienced acute vomiting within 1 hour of taking primaquine between days 0 and 14**

| **Primaquine daily dose** | **Days of observation** |
| --- | --- |
|  | **0–14** |
| Low | 0/206 (0%) |
| Intermediate | 15/642 (2·3%) |
| High | 14/380 (3·7%) |
| Total | 29/1228 (2·4%) |


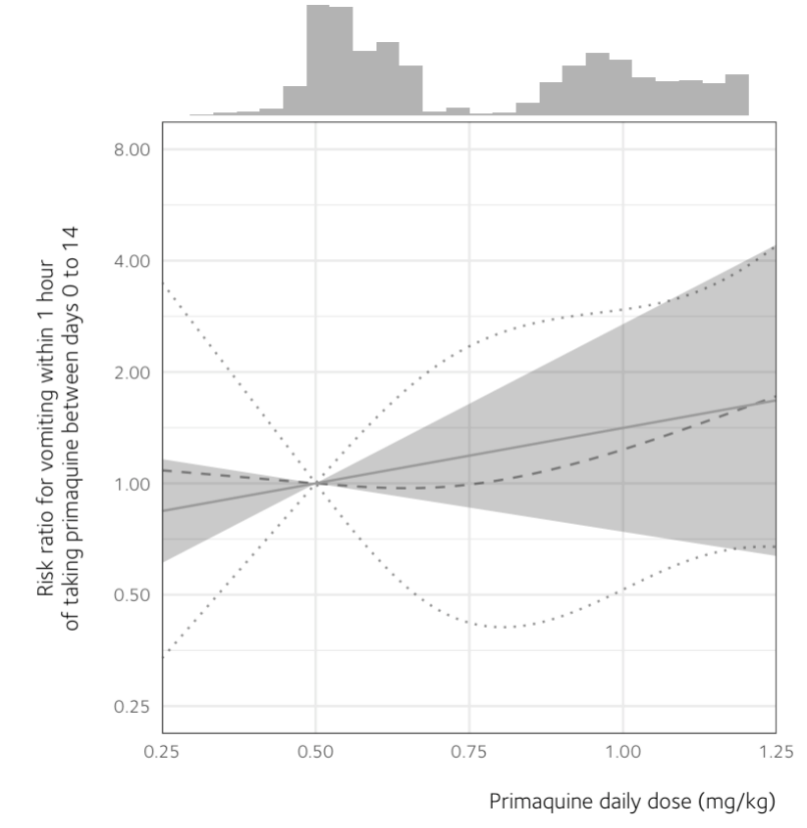


**Figure S16. Risk ratio of acute vomiting within 1 hour of taking primaquine between days 0 and 14**

The reference value, where the risk ratio equals one, was set at the primaquine daily dose of 0·5 mg/kg. Estimates were derived from a multivariable Poisson model (n = 767). Two different specifications for primaquine daily dose were shown to evaluate potential non-linearity across doses: trend (solid grey line with shaded region for 95% CI) and restricted cubic spline with three knots (dashed curve with two dotted curves representing the 95% CI limits). The histogram along the top margin shows the distribution of primaquine daily doses in the model data. The vertical axis is shown on a logarithmic scale.

**Table S10. Poisson model estimates with robust standard errors**

| **Model** | **RR (95% robust CI)** |
| --- | --- |
| **Tolerability** | |
| Effect of increasing 0·25 mg/kg daily dose on GI discomfort (days 5–7) | 1·32 (1·17 to 1·48) |
| Effect of increasing 0·25 mg/kg daily dose on GI discomfort (day 0) | 0·99 (0·96 to 1·02) |
| Effect of increasing 0·25 mg/kg daily dose on GI discomfort (days 1–2) | 1·03 (0·97 to 1·10) |
| Effect of increasing 0·25 mg/kg daily dose on acute vomiting | 1·19 (0·84 to 1·67) |
| **Safety** | |
| Effect of increasing 0·25 mg/kg daily dose on clinical anaemia | 0·90 (0·80 to 1·01) |


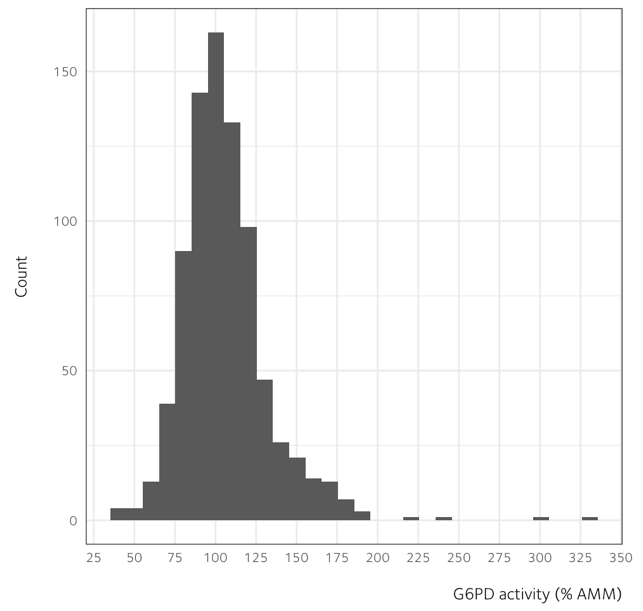

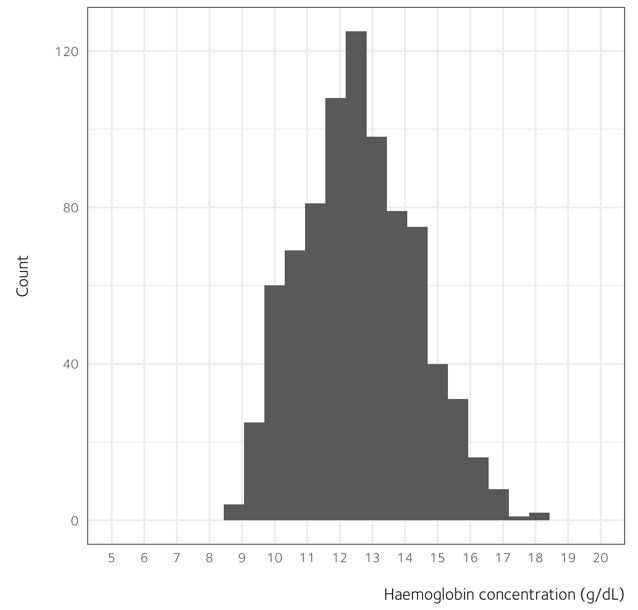


A

B

**Figure S17. (A) Haemoglobin concentrations and (B) G6PD activity levels at patient enrolment in the primary safety dataset (n = 822)**

**Table S11. Haematological adverse events on days 1–14, by dose group**

| **Haematological adverse event** | **Primaquine daily dose** | | | |
| --- | --- | --- | --- | --- |
|  | **No primaquine** | **Low** | **Intermediate** | **High** |
| Haemoglobin fall to <5 g/dL | 0/173 | 0/6 | 0/318 | 0/325 |
| Haemoglobin fall >5 g/dL from baseline | 0/173 | 0/6 | 0/318 | 0/325 |
| Blood transfusion | 0/173 | 0/6 | 0/318 | 0/325 |
| Renal failure requiring dialysis | 0/173 | 0/6 | 0/318 | 0/325 |
| Death | 0/173 | 0/6 | 0/318 | 0/325 |


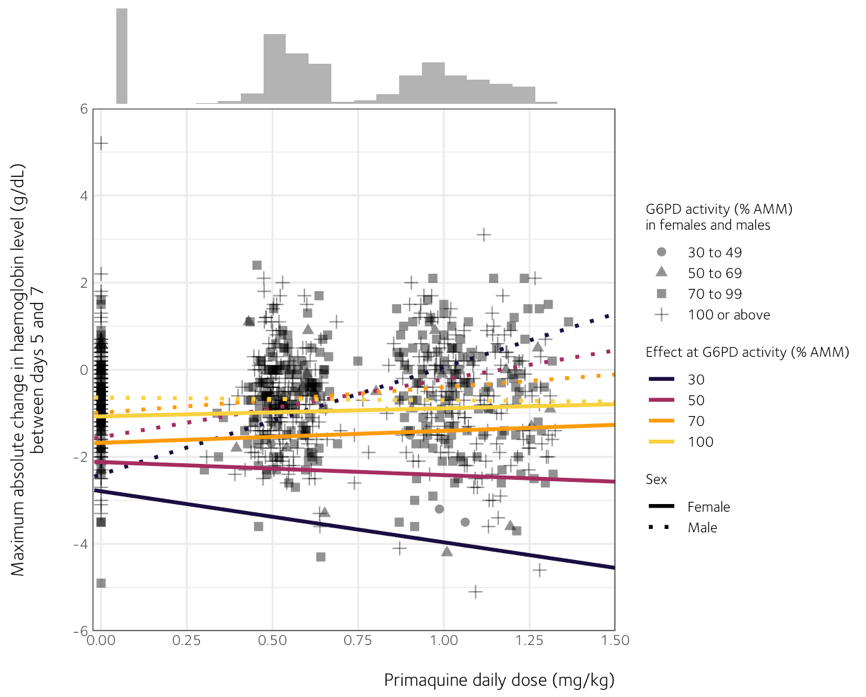


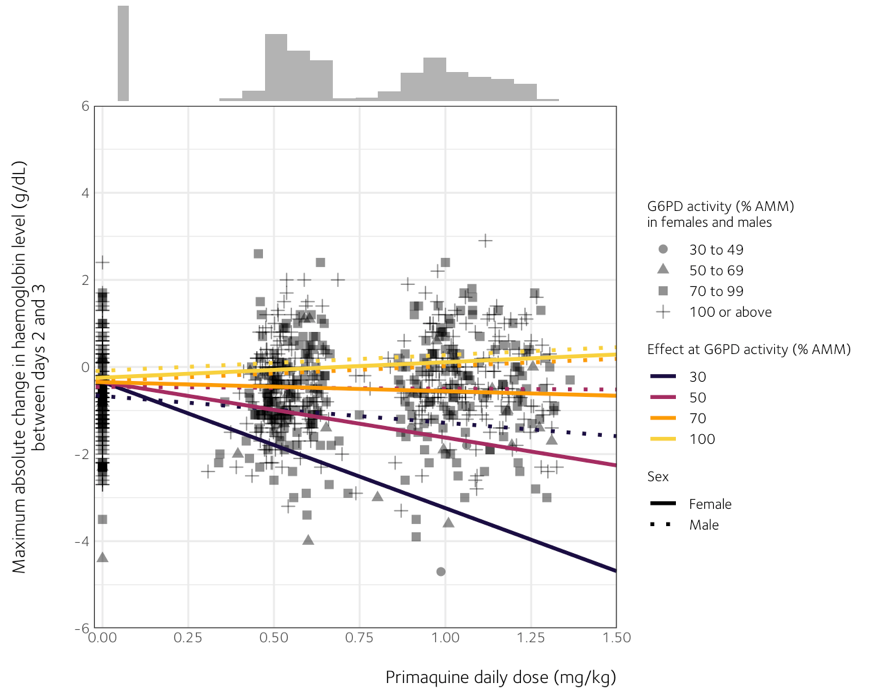


B

AB

**Figure S18. Maximum absolute change from baseline in haemoglobin levels (A) between days 2 and 3 and (B) between days 5 and 7 across primaquine daily doses, sex, and G6PD activity levels**

Estimates were derived from a multivariable linear model fitted to the subsets of the primary safety dataset (n in panel A = 794, n in panel B = 781). An interaction term was included in the model to account for differential treatment effects of primaquine daily dose as sex and G6PD activity vary. A restricted cubic spline was specified for G6PD activity to model a non-linear trend. Primaquine daily dose and G6PD activity were both modelled as continuous covariates. The plot shows model-implied predictions only at four selected cut-offs (30%, 50%, 70%, and 100%) of G6PD activity to aid interpretation. The histogram along the top margin shows the distribution of primaquine daily doses in the model data, with the leftmost bar representing patients who were treated without primaquine (i.e., 0 mg/kg). The vertical axis is shown on a logarithmic scale. AMM, adjusted male median.


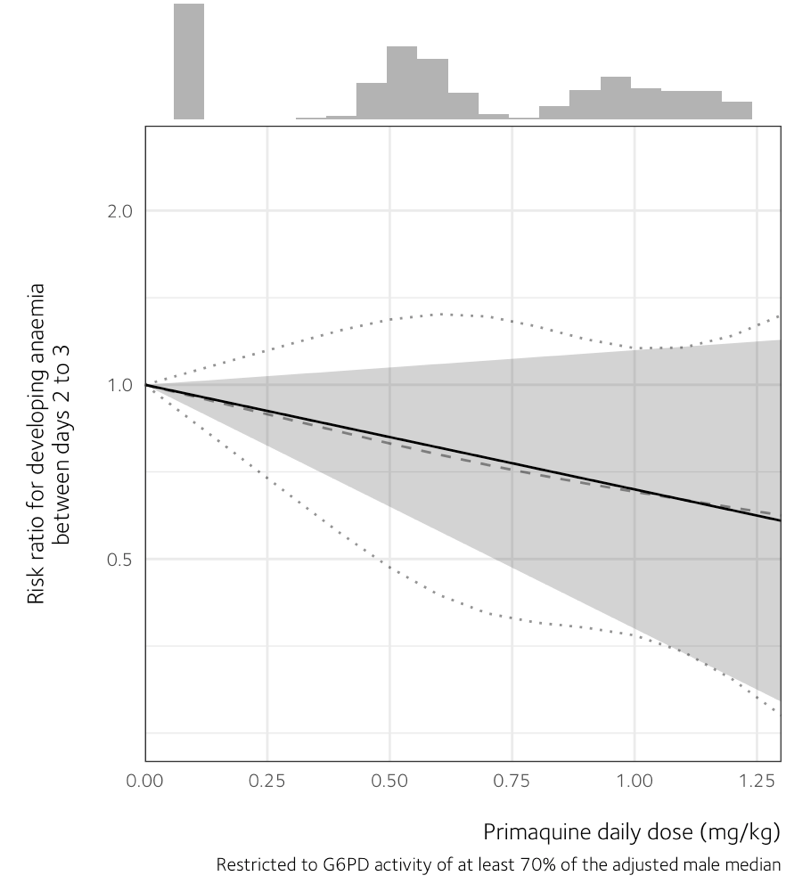


**Figure S19. Risk ratio of developing anaemia (haemoglobin <11 g/dL) between days 2 to 3 in patients with G6PD activity of at least 70% and had baseline haemoglobin level of at least 11 g/dL**

The reference value, where the risk ratio equals one, was set at the primaquine daily dose of 0 mg/kg. Estimates were derived from a multivariable Poisson model, fitted to a subset of the primary tolerability dataset. Two different specifications for primaquine daily dose were shown to evaluate potential non-linearity across doses: linear trend (solid grey line with shaded region for 95% CI) and restricted cubic spline with three knots (dashed curve with two dotted curves representing the 95% CI limits). The histogram along the top margin shows the distribution of primaquine daily doses in the model data (n = 612) , with the leftmost bar representing patients who were treated without primaquine (i.e., 0 mg/kg). The vertical axis is shown on a logarithmic scale.


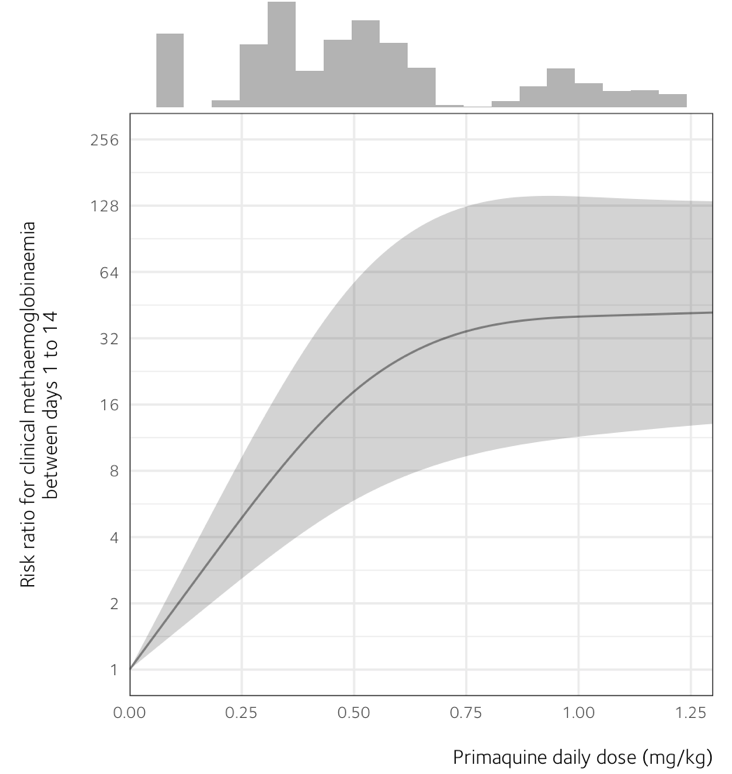


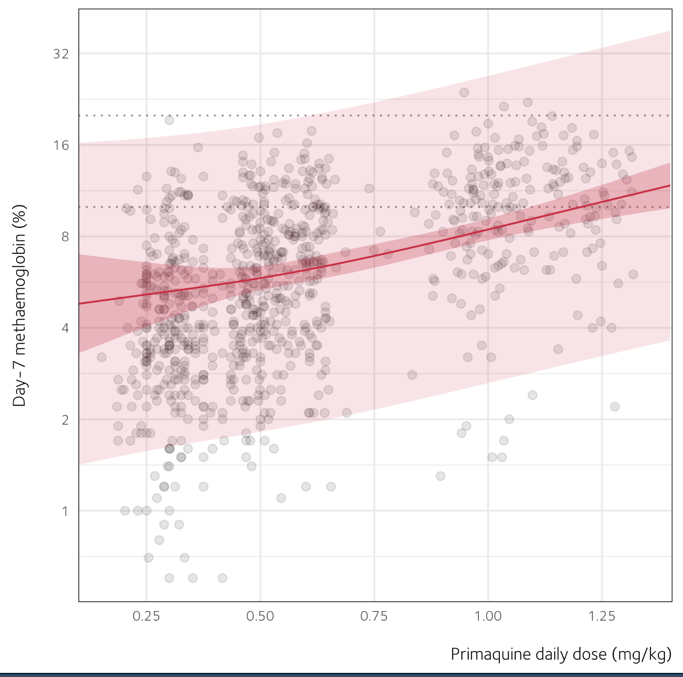


B

A

**Figure S20. Effect of primaquine daily dose on methaemoglobin levels**

(A) Effect of primaquine daily dose on day 7 methaemoglobin levels (n = 886), modelled using multivariable linear regression, adjusting for age, sex, baseline parasite density, and study site. A restricted cubic spline with three knots was applied to primaquine daily dose. The solid curve represents the expected day 7 methaemoglobin levels across the range of primaquine daily doses. The thick and thin shaded regions represent the 95% confidence intervals and prediction intervals, respectively. Each dot represents an individual patient's day 7 methaemoglobin level. The dotted horizontal lines have y-intercepts of 10% and 20%, thresholds at which symptoms related to tissue hypoxia (e.g., light-headedness, tachycardia) may become more apparent. (B) Risk ratios of clinical methaemoglobinaemia (i.e., 10% or more) from day 1 to 14 across primaquine daily doses. Estimates were modelled using a multivariable Poisson model with a restricted cubic spline with three knots applied to primaquine daily dose. The solid curve and shaded region represent the point estimates and 95% confidence intervals, respectively. The histogram along the top margin shows the distribution of primaquine daily doses in the model data (n = 1026) , with the leftmost bar representing patients who were treated without primaquine (i.e., 0 mg/kg). The vertical axis is shown on a logarithmic scale.

References

1. Price RN, Commons RJ, Battle KE, Thriemer K, Mendis K. *Plasmodium vivax* in the era of the shrinking *P. falciparum* map. *Trends Parasitol* 2020; **36**(6): 560-70.

2. Kementerian Kesehatan Republik Indonesia. Laporan situasi terkini perkembangan program pengendalian malaria di Indonesia tahun 2024, 2025. https://malaria.kemkes.go.id/sites/default/files/2025-05/Factsheet%202024.pdf (accessed: 18/10/2025).

3. Djaafara BA, Sherrard-Smith E, Churcher TS, et al. Spatiotemporal heterogeneity in malaria transmission across Indonesia: analysis of routine surveillance data 2010–2019. *BMC Med* 2025; **23**(1): 136.

4. Fadilah I, Djaafara BA, Lestari KD, et al. Quantifying spatial heterogeneity of malaria in the endemic Papua region of Indonesia: Analysis of epidemiological surveillance data. *Lancet Reg Health Southeast Asia* 2022; **5**.

5. Phyo AP, Dahal P, Mayxay M, Ashley EA. Clinical impact of vivax malaria: a collection review. *PLoS Med* 2022; **19**(1): e1003890.

6. Commons RJ, Rajasekhar M, Edler P, et al. Effect of primaquine dose on the risk of recurrence in patients with uncomplicated *Plasmodium vivax*: a systematic review and individual patient data meta-analysis. *Lancet Infect Dis* 2023.

7. Rajasekhar M, Simpson JA, Ley B, et al. Primaquine dose and the risk of haemolysis in patients with uncomplicated *Plasmodium vivax* malaria: a systematic review and individual patient data meta-analysis. *Lancet Infect Dis* 2023.

8. World Health Organization. WHO guidelines for malaria, 30 November 2024, 2024. https://www.who.int/publications/i/item/guidelines-for-malaria (accessed: 18/10/2025).

9. Kementerian Kesehatan Republik Indonesia. Buku saku tatalaksana kasus malaria; 2023. https://malaria.kemkes.go.id/sites/default/files/2024-02/X_Cetak%20Buku%20Saku%20talak%20Des%202023F.pdf (accessed: 18/10/2025).

10. Sadhewa A, Cassidy-Seyoum S, Acharya S, et al. A review of the current status of G6PD deficiency testing to guide radical cure treatment for vivax malaria. *Pathogens* 2023; **12**(5): 650.

11. Nelwan EJ, Ekawati LL, Tjahjono B, et al. Randomized trial of primaquine hypnozoitocidal efficacy when administered with artemisinin-combined blood schizontocides for radical cure of *Plasmodium vivax* in Indonesia. *BMC Med* 2015; **13**: 294.

12. Poespoprodjo JR, Burdam FH, Candrawati F, et al. Supervised versus unsupervised primaquine radical cure for the treatment of falciparum and vivax malaria in Papua, Indonesia: a cluster-randomised, controlled, open-label superiority trial. *Lancet Infect Dis* 2022; **22**(3): 367-76.

13. Sutanto I, Tjahjono B, Basri H, et al. Randomized, Open-Label Trial of Primaquine against Vivax Malaria Relapse in Indonesia. *Antimicrob Agents Chemother* 2013; **57**(3): 1128-35.

14. Pasaribu AP, Chokejindachai W, Sirivichayakul C, et al. A randomized comparison of dihydroartemisinin-piperaquine and artesunate-amodiaquine combined with primaquine for radical treatment of vivax malaria in Sumatera, Indonesia. *J Infect Dis* 2013; **208**(11): 1906-13.

15. Taylor WRJ, Thriemer K, Von Seidlein L, et al. Short-course primaquine for the radical cure of *Plasmodium vivax* malaria: a multicentre, randomised, placebo-controlled non-inferiority trial. *Lancet* 2019; **394**(10202): 929-38.

16. Commons RJ, Thriemer K, Humphreys G, et al. The Vivax Surveyor: Online mapping database for *Plasmodium vivax* clinical trials. *Int J Parasitol Drugs Drug Resist* 2017; **7**(2): 181-90.

17. Infectious Diseases Data Observatory (IDDO). IDDO SDTM implementation manual. 2023. <https://www.iddo.org/tools-and-resources/data-tools> (accessed: 01/04/2024).

18. Stewart LA, Clarke M, Rovers M, et al. Preferred Reporting Items for Systematic Review and Meta-Analyses of individual participant data: the PRISMA-IPD Statement. *JAMA* 2015; **313**(16): 1657-65.

19. World Health Organization. Technical consultation to review the classification of glucose-6-phosphate dehydrogenase (G6PD), 2022. https://cdn.who.int/media/docs/default-source/malaria/mpac-documentation/mpag-mar2022-session2-technical-consultation-g6pd-classification.pdf?sfvrsn=1f36be5e_7&download=true (accessed: 18/10/2025).

20. Zou G. A modified poisson regression approach to prospective studies with binary data. *American journal of epidemiology* 2004; **159**(7): 702-6.

21. Sterne JA, Savović J, Page MJ, et al. RoB 2: a revised tool for assessing risk of bias in randomised trials. *BMJ* 2019; **366**.

22. Sterne JA, Hernán MA, Reeves BC, et al. ROBINS-I: a tool for assessing risk of bias in non-randomised studies of interventions. *BMJ* 2016; **355**.

23. WorldWide Antimalarial Resistance Network (WWARN). Primaquine Indonesia study group. 2023. [https://www.iddo.org/primaquine-indonesia-study-group (accessed](https://www.iddo.org/primaquine-indonesia-study-groupaccessed): 27/02/2025).

24. Hasugian A, Purba H, Kenangalem E, et al. Dihydroartemisinin-piperaquine versus artesunate-amodiaquine: superior efficacy and posttreatment prophylaxis against multidrug-resistant *Plasmodium falciparum* and *Plasmodium vivax* malaria. *Clin Infect Dis* 2007; **44**(8): 1067-74.

25. Lidia K, Dwiprahasto I, Kristin E. Therapeutic Effects of Dyhidroartemisinin Piperaquine Versus Chloroquine for Uncomplicated Vivax Malaria in Kupang, East Nusa Tenggara, Indonesia. *Age* 2015; **13**: 50.

26. Sutanto I, Soebandrio A, Ekawati LL, et al. Tafenoquine co-administered with dihydroartemisinin-piperaquine for the radical cure of *Plasmodium vivax* malaria (INSPECTOR): a randomised, placebo-controlled, efficacy and safety study. *Lancet Infect Dis* 2023; 1;23(10):1153-63.

27. Maguire JD, Krisin, Marwoto H, Richie TL, Fryauff DJ, Baird JK. Mefloquine is highly efficacious against chloroquine-resistant *Plasmodium vivax* malaria and Plasmodium falciparum malaria in Papua, Indonesia. *Clin Infect Dis* 2006; **42**(8): 1067-72.

28. Arcelia F, Pasaribu AP, Yanni GN. Effectiveness of dihydroartemisinin-piperaquine after 10 years as treatment for vivax malaria in Indonesia. *J Infect Dev Ctries* 2023; **17**(05): 700-6.

29. Chu CS, Bancone G, Moore KA, et al. Haemolysis in G6PD heterozygous females treated with primaquine for *Plasmodium vivax* malaria: a nested cohort in a trial of radical curative regimens. *PLoS Med* 2017; **14**(2): e1002224.

30. Lipsitch M, Tchetgen ET, Cohen T. Negative controls: a tool for detecting confounding and bias in observational studies. *Epidemiol* 2010; **21**(3): 383-8.

31. Rajgor D, Gogtay N, Kadam V, et al. Antirelapse efficacy of various primaquine regimens for *Plasmodium vivax*. *Malar Res Treat* 2014; **2014**(1): 347018.

32. Saravu K, Tellapragada C, Kulavalli S, et al. A pilot randomized controlled trial to compare the effectiveness of two 14-day primaquine regimens for the radical cure of vivax malaria in South India. *Malar J* 2018; **17**: 1-11.

33. Chamma-Siqueira NN, Negreiros SC, Ballard S-B, et al. Higher-dose primaquine to prevent relapse of *Plasmodium vivax* malaria. *N Engl J Med* 2022; **386**(13): 1244-53.

34. Eng V, Lek D, Sin S, et al. 14 days of high-dose versus low-dose primaquine treatment in patients with *Plasmodium vivax* infection in Cambodia: a randomised, single-centre, open-label efficacy study. *Lancet Infect Dis* 2025.

35. Degaga TS, Pasaribu AP, Tripura R, et al. Effectiveness and safety of high dose primaquine and tafenoquine in Plasmodium vivax patients (EFFORT)-a multi-centre, open label, superiority randomised controlled trial. *Lancet Infect Dis* 2026; 0.

36. Collins WE, Jeffery GM. Primaquine resistance in *Plasmodium vivax*. *Am J Trop Med Hyg* 1996; **55**(3): 243-9.

37. Garrison PL, Hankey DD, Coker WG, et al. 2. CURE OF KOREAN VIVAX MALARIA WITH PAMAQUINE AND PRIMAQUINE. *J Am Med Assoc* 1952; **149**(17): 1562-3.

38. White NJ. Determinants of relapse periodicity in *Plasmodium vivax* malaria. *Malar J* 2011; **10**(1): 297.

39. Lestari KD, Surendra H, Djaafara BA, et al. Epidemiology of malaria and district-level factors associated with malaria elimination in Sumatra region, Indonesia: a retrospective analysis of surveillance data. *medRxiv* 2025: 2025.08. 28.25333185. https://www.medrxiv.org/content/10.1101/2025.08.28.25333185v1 (accessed: 18/10/2025).

40. Mehdipour P, Rajasekhar M, Dini S, et al. Effect of adherence to primaquine on the risk of *Plasmodium vivax* recurrence: a WorldWide Antimalarial Resistance Network systematic review and individual patient data meta-analysis. *Clin Infect Dis* 2023; **22**(1): 306.

41. Fryauff D, Baird K, Basri H, et al. Randomised placebo-controlled trial of primaquine for prophylaxis of falciparum and vivax malaria. *Lancet* 1995; **346**(8984): 1190-3.

42. Clayman CB, Arnold J, Hockwald RS, Yount EH, Edgcomb JH, Alving AS. 3. Toxicity of primaquine in caucasians. *J Am Med Assoc* 1952; **149**(17): 1563-8.

43. Baird JK, Hoffman SL. Primaquine therapy for malaria. *Clin Infect Dis* 2004; **39**(9): 1336-45.

44. Fadilah I, Commons RJ, Chau NH, et al. Methaemoglobin as a surrogate marker of primaquine antihypnozoite activity in *Plasmodium vivax* malaria: A systematic review and individual patient data meta-analysis. *PLoS Med* 2024; **21**(9): e1004411.

45. White NJ, Watson JA, Baird JK. Methaemoglobinaemia and the radical curative efficacy of 8-aminoquinoline antimalarials. *Br J Clin Pharmacol* 2022; **88**(6): 2657-64.

46. Watson JA, Commons RJ, Tarning J, et al. The clinical pharmacology of tafenoquine in the radical cure of *Plasmodium vivax* malaria: An individual patient data meta-analysis. *Elife* 2022; **11**.

47. Green JA, Mohamed K, Goyal N, et al. Pharmacokinetic interactions between tafenoquine and dihydroartemisinin-piperaquine or artemether-lumefantrine in healthy adult subjects. *Antimicrob Agents Chemother* 2016; **60**(12): 7321-32.

48. Commons RJ, Simpson JA, Watson J, White NJ, Price RN. Estimating the Proportion of *Plasmodium vivax* Recurrences Caused by Relapse: A Systematic Review and Meta-Analysis. *Am J Trop Med Hyg* 2020; **103**(3): 1094-9.

49. Battle KE, Lucas TCD, Nguyen M, et al. Mapping the global endemicity and clinical burden of *Plasmodium vivax*, 2000–17: a spatial and temporal modelling study. *Lancet* 2019; **394**(10195): 332-43.

50. Becker RAW, A. R.; Brownrigg, R.; Minka, T. P.; Deckmyn, A. maps: Draw Geographical Maps. 3.4.1 ed: R package; 2023.

51. Wickham HC, W.; Henry, L.; Pedersen, T. L.; Takahashi, K.; Wilke, C.; Woo, K.; Yutani, H. ggplot2: Create Elegant Data Visualisations Using the Grammar of Graphics. 3.5.1 ed: R package; 2023.

52. Battle KE, Karhunen MS, Bhatt S, et al. Geographical variation in *Plasmodium vivax* relapse. *Malar J* 2014; **13**(1): 144.
